# Arrhythmogenic Hearts in PKD2 Mutant Mice Are Characterized by Cardiac Fibrosis, Systolic, and Diastolic Dysfunctions

**DOI:** 10.3389/fcvm.2021.772961

**Published:** 2021-11-26

**Authors:** Farideh Amirrad, Rajasekharreddy Pala, Kiumars Shamloo, Brian S. Muntean, Surya M. Nauli

**Affiliations:** ^1^Department of Biomedical and Pharmaceutical Sciences, Chapman University, Irvine, CA, United States; ^2^Department of Medicine, University of California, Irvine, Orange, CA, United States; ^3^Department of Pharmacology and Toxicology, Medical College of Georgia, Augusta University, Augusta, GA, United States

**Keywords:** polycystic kidney disease, cardiovascular, fibrosis, cardiac function, inflammation

## Abstract

Autosomal dominant polycystic kidney disease (PKD) is a hereditary disorder affecting multiple organs, including the heart. PKD has been associated with many cardiac abnormalities including the arrhythmogenic remodeling in clinical evaluations. In our current study, we hypothesized that *Pkd2* gene mutation results in structural and functional defects in the myocardium. The structural and functional changes of *Pkd2* mutant hearts were analyzed in the myocardial-specific *Pkd2* knockout (KO) mouse. We further assessed a potential role of TGF-b_1_ signaling in the pathology of *Pkd2*-KO hearts. Hearts from age-matched 6-month-old *MyH6*•*Pkd2*^*wt*/*wt*^ (control or wild-type) and *MyH6*•*Pkd2*^*flox*/*flox*^ (mutant or *Pkd2*-KO) mice were used to study differential heart structure and function. Cardiac histology was used to study structure, and the “isolated working heart” system was adapted to mount and perfuse mouse heart to measure different cardiac parameters. We found that macrophage1 (M1) and macrophage 2 (M2) infiltration, transforming growth factor (TGF-b_1_) and TGF-b_1_ receptor expressions were significantly higher in *Pkd2*-KO, compared to wild-type hearts. The increase in the extracellular matrix in *Pkd2*-KO myocardium led to cardiac hypertrophy, interstitial and conduction system fibrosis, causing cardiac dysfunction with a predisposition to arrhythmia. Left ventricular (LV) expansion or compliance and LV filling were impaired in fibrotic *Pkd2*-KO hearts, resulted in diastolic dysfunction. LV systolic contractility and elastance decreased in fibrotic *Pkd2*-KO hearts, resulted in systolic dysfunction. Compared to wild-type hearts, *Pkd2*-KO hearts were less responsive to the pharmacological stress-test and changes in preload. In conclusion, *Pkd2*-KO mice had systolic and diastolic dysfunction with arrhythmogenic hearts.

## Introduction

Autosomal dominant polycystic kidney disease (PKD) is the most common hereditary renal disorder that affects 1 in 800 live births. Mutations in the *Pkd1* and *Pkd2* genes that encode membrane-associated polycystin-1 and polycystin-2, respectively, contribute to the development of cystic kidneys, which are characterized with severe renal fibrosis (1–3). Polycystin-1 is an eleven-transmembrane protein interacting with polycystin-2, a member of the transient receptor potential (TRP) protein family that forms a calcium-permeable cation channel. Polycystin-2 is a non-selective cation channel with high calcium (Ca^2+^) permeability. The genetic mutation eventually causes kidney enlargement, deformation, renal failure, and various extra-renal manifestations (4–6).

Cardiovascular complications are a major cause of morbidity and mortality in PKD patients. Cardiovascular manifestations in PKD include hypertension (7, 8), left ventricular hypertrophy (7, 9), valvular heart disease (10), intracranial and extracranial aneurysms (11), and atrial fibrillation (11). Hypertension occurs in 50–70% of patients before any significant depletion in the glomerular filtration rate, and it occurs at an earlier age in PKD patients compared to the general population (8). Hypertension is a main early finding of PKD prior to any renal dysfunction, which can accelerate end-stage kidney disease in about 60 percent of PKD patients (12, 13).

The cardiovascular complications in PKD, including hypertension and cardiac dysfunction, are thought to be secondary to polycystic kidneys. However, recent studies indicate that alterations in the polycystins expression directly affect the cardiomyocyte, endothelial and vascular smooth muscles functions, which could be responsible for cardiovascular disease (14). Polycystin-1 and−2 have been proposed to function as pressure sensors within the cardiovascular system (15). Polycystin-1 and−2 function as mechanosensory proteins in the cardiomyocytes to govern cardiomyocyte contractility (16–18). Both polycystins play a role in intracellular calcium homeostasis by interacting with the ryanodine receptor, which induces calcium release from the endoplasmic reticulum in the heart (19).

Left ventricular hypertrophy (LVH) is another cardiovascular complication commonly measured in clinical studies using echocardiography in PKD patients (9, 20). LVH is a significant and independent risk factor for cardiovascular morbidity and mortality, and it is associated with poor prognosis in PKD patients. The presence of LVH affects clinical outcomes, with increased risk of atrial or ventricular arrhythmia (21), systolic and diastolic dysfunction (22, 23), congestive heart failure (24), cardiovascular mortality (21), and sudden cardiac death (21). LVH has a high prevalence in PKD patients with hypertension (7) or even in non-hypertensive PKD patients (25). In extensive echocardiographic analyses, LVH is predicted to be associated with left ventricular fibrosis (9, 26). Cardiac fibrosis is a scarring process in the cardiac muscle with collagen deposition, fibroblast activation, and fibroblasts differentiation to myofibroblasts (27).

Our current studies examined the hypothesis that *Pkd2* gene mutation resulted in structural and functional heart defects. We studied fibrosis and fibrotic pathways in the cardiac tissues of *Pkd2* mutant mouse model, and the effect of *Pkd2* gene mutation on cardiac function was studied using an isolated working heart apparatus.

## Results

To identify any abnormality in the structure and function of PKD hearts, transgenic heart-specific mice were used throughout the studies unless indicated. The *MyH6*•*Pkd2 mutant*^*wt*/*wt*^ mice were used as control or wild-type (WT), and *MyH6*•*Pkd2 mutant*^*flox*/*flox*^ was designated as mutant (*Pkd2*-KO).

### The *Pkd2*-KO Hearts Were Characterized With Hypertrophy, Cardiac Fibrosis, and Fibrotic Conduction System

**Figure 1:**
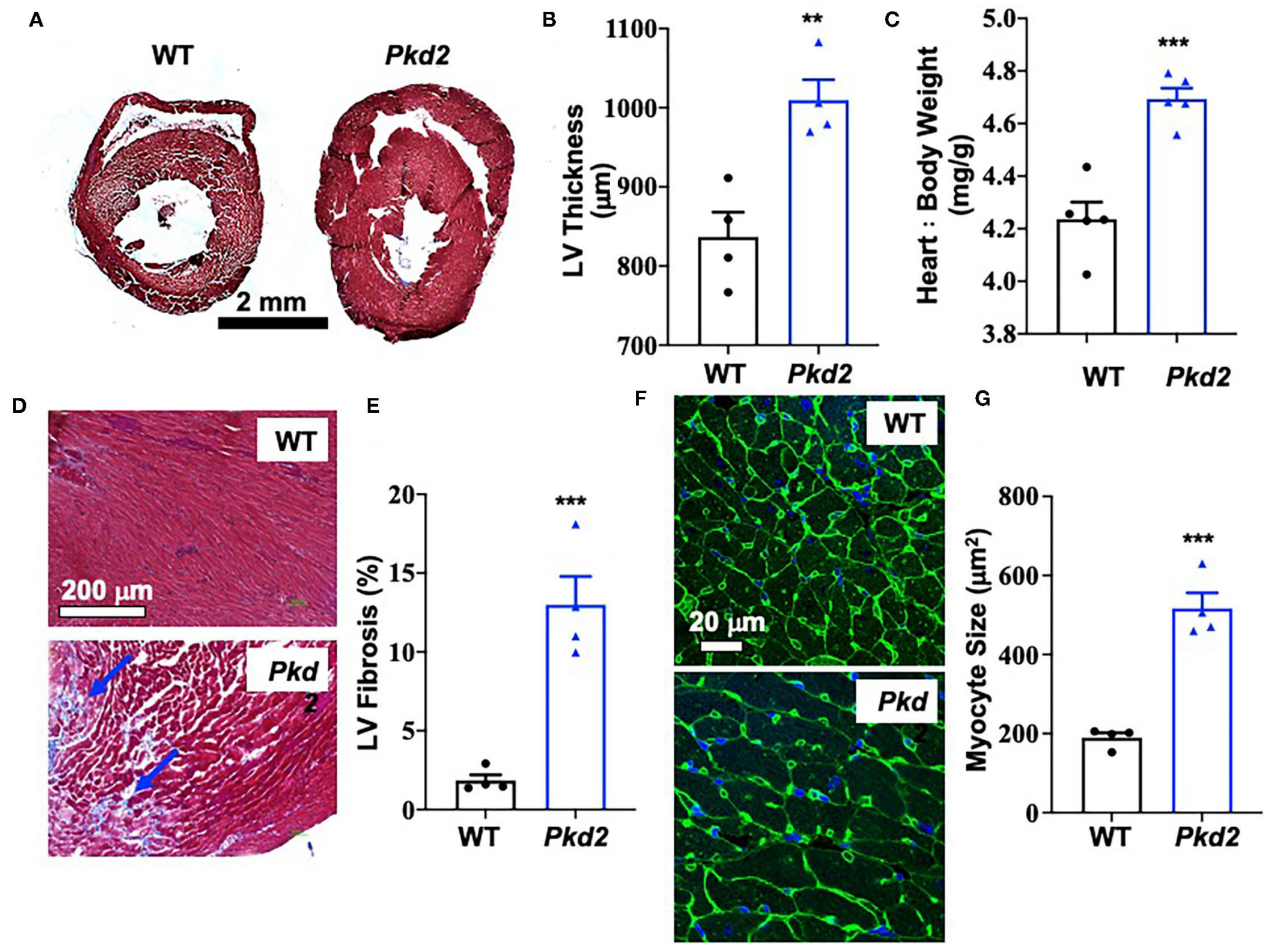
The hearts from *Pkd2*-KO mice are characterized with hypertrophy and fibrosis. **(A–C)** Masson's trichrome staining was used to analyzed hearts from wild-type (WT) and *Pkd2*-KO (*Pkd2*) mice. Representative of left ventricular thickness (hypertrophy) is shown **(A)** and quantified **(B,C)**. **(D,E)** Left ventricular (LV) fibrosis is analyzed; arrows indicate fibrosis **(D)**. LV fibrosis was quantified **(E)**. Data show a significant increase in LV myocardial hypertrophy and fibrosis in *Pkd2*-KO compared to WT mice. Fibrosis revealed by trichrome cooperation in the tissue was quantified with binary masking of the blue-color image **(D,E)**. WGA staining was used for cell surface staining and cell size measurement. Representative of LV cardiomyocyte size (hypertrophy) is shown **(F)** and quantified **(G)**. *N* = 4 mice for each WT and *Pkd2*-KO group at age matched of 6 months old. **p < 0.01; ***p < 0.001.

**Figure 2:**
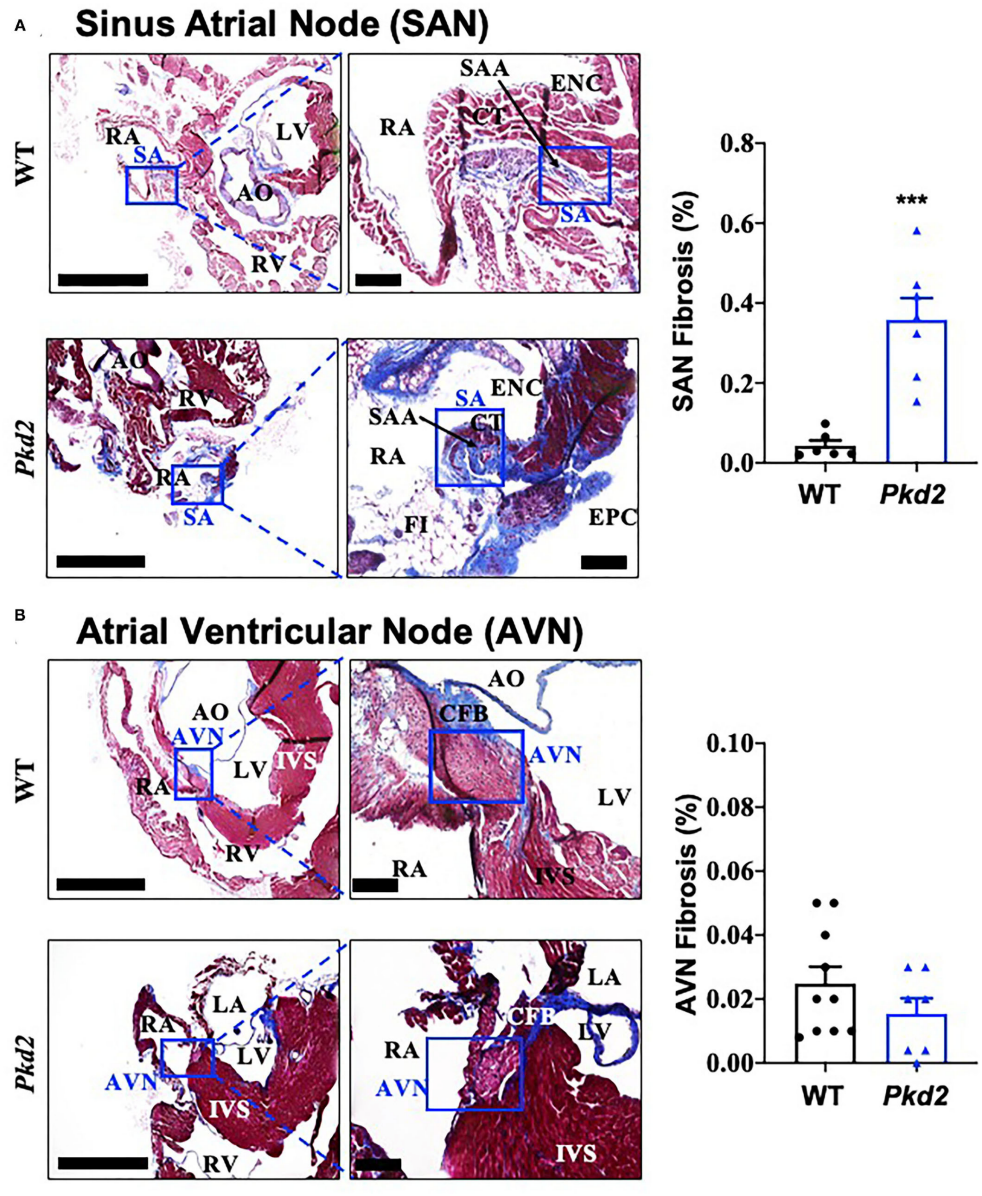
The sinoatrial node (SAN) *Pkd2*-KO mice are characterized with fibrosis and fatty infiltration. Masson's trichrome staining was performed in wild-type (WT) and *Pkd2*-KO (*Pkd2*) mouse hearts. **(A)** A significant increase of fibrotic tissue is seen in SAN of right atrium in PKD mouse compared to WT. Boxes indicated SAN, and the left panel is further magnified and shown in the right panel. **(B)** No significant difference of fibrotic tissue is detected in AVN of right atrium in *Pkd2*-KO mouse. Boxes indicated AVN, and the left panel is further magnified and shown in the right panel. AO, aorta; AVN, atrioventricular node; CFB, central fibrous body; CT, Crista terminalis; ENC, endocardium; EPC, epicardium; FI, fatty infiltration; IVS, interventricular septum; LV, left ventricle; RA, right atrium; RV, right ventricle; SA, sinoatrial node; SAA, sinoatrial artery. Scale Bar = 20 mm. *N* = 6–10 mice for each WT and *Pkd2*-KO group at age matched of 6 months old. ***p < 0.001.

**Figure 3:**
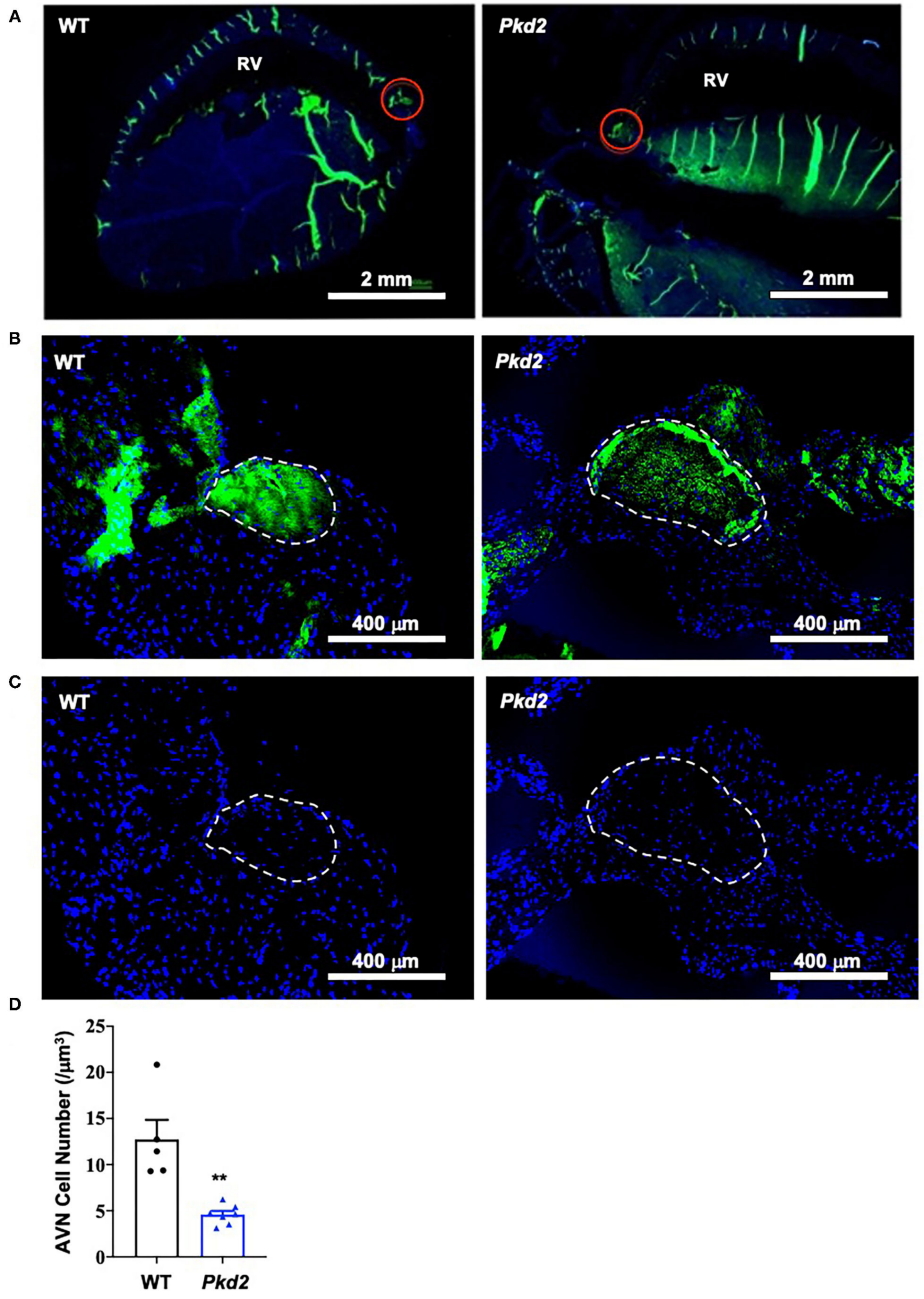
Cell number is decreased in *Pkd2*-KO mouse atrioventricular node. **(A)** Heart tissues were stained with anti-HCN4 antibody used as a marker for the myocardial conduction system (green staining). Atrioventricular node (AVN) is identified by both location and HCN4-postive staining indicated by the red circles; RV, right ventricle. **(B)** The AVN is next identified as region of interest. **(C)** Number of cell nucleus (DAPI; blue staining) is counted. **(D)** Data reveal a significantly lower number of cells in AVN of *Pkd2*-KO mouse hearts. *N* = 5 mice for each WT and *Pkd2*-KO group at age matched of 6 months old. **p < 0.01.

The most apparent phenotype in the *Pkd2*-KO hearts was hypertrophy and interstitial fibrosis. The *Pkd2*-KO left ventricular (LV) myocardial thickness (Figures 1A,B) and heart-to-body-weight ratio (Figure 1C) were significantly increased in *Pkd2*-KO compared to control WT hearts. The extensive diffuse and reactive interstitial fibrosis of LV were significantly higher in *Pkd2*-KO, compared to WT hearts (Figures 1D,E). The size of LV myocardiocytes was significantly larger in *Pkd2*-KO than WT hearts (Figures 1F,G), supporting the myocyte hypertrophy in *Pkd2*-KO hearts. The sinoatrial node (SAN) and atrioventricular node (AVN) were also studied to examine fibrosis in the cardiac conduction system in *Pkd2*-KO mice. Upregulation of fatty and fibrotic tissues was observed in SAN and surrounding tissues in *Pkd2*-KO mice (Figure 2A). Although significant fibrosis was detected in SAN, fibrosis was not statistically significant in AVN between *Pkd2*-KO and WT hearts (Figure 2B). Because no significant fibrosis was found in AVN, cell numbers were further analyzed (Figure 3). AVN was identified by both its position within the heart and localization of hyperpolarization-activated cyclic nucleotide-gated channel 4 (HCN4), which is known to localize in heart conduction systems. AVN significantly had a lower cell number in *Pkd2*-KO than WT hearts.

**Figure 4:**
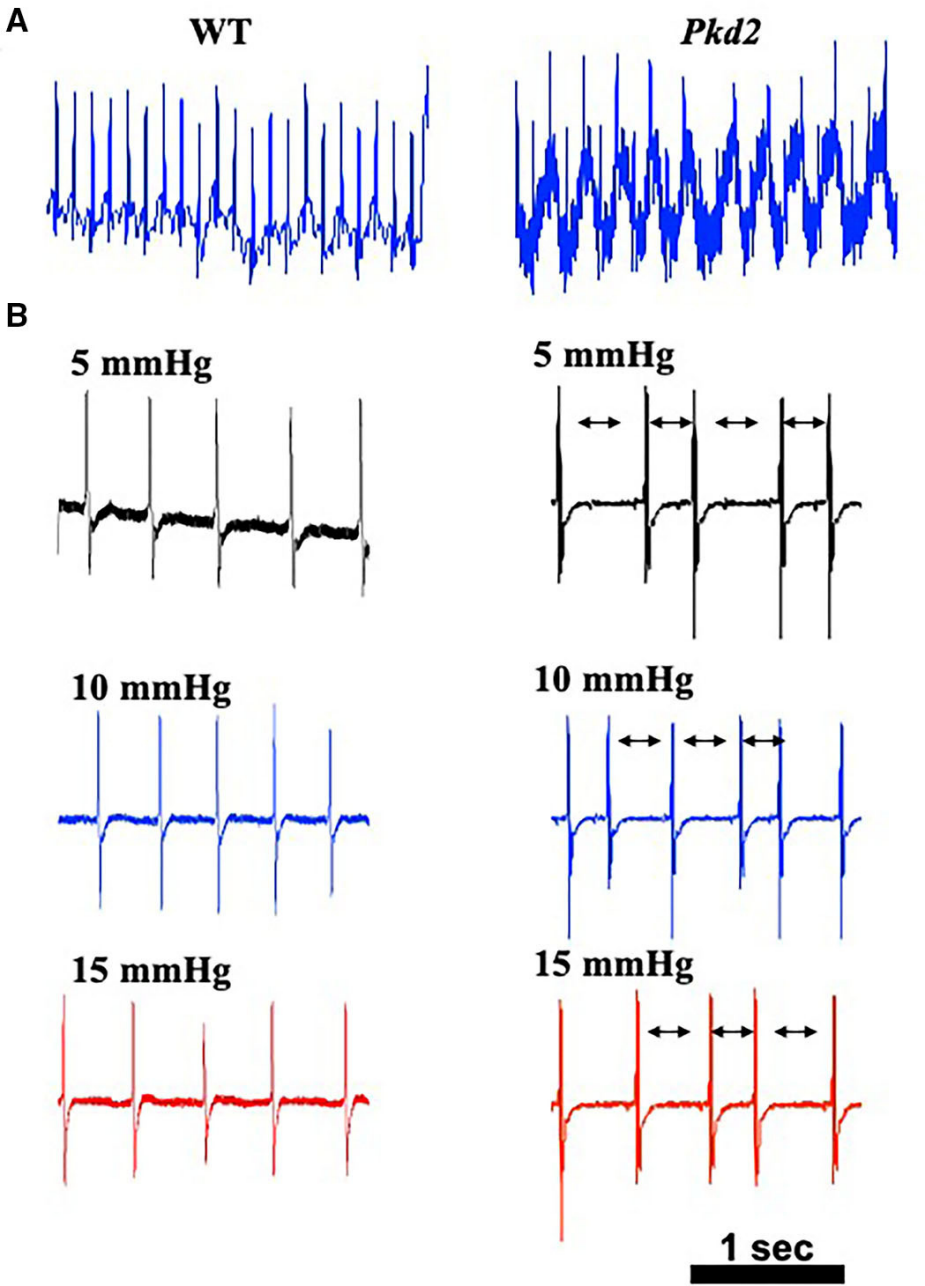
The hearts from *Pkd2*-KO mice with no autonomic input have irregular beats. **(A)** Representatives of *in vivo* electrocardiogram (ECG) are shown with averaged frequency of 540–600 beats/min in wild-type (WT) and *Pkd2*-KO (*Pkd2*) mice. **(B)** Representatives of *ex vivo* ECG with no autonomic input are shown with different preloads of 5, 10, and 15 mmHg. The *Pkd2*-KO heart ECG shows irregular heartbeat with normal QRS shape. All ECG from *Pkd2*-KO showed irregular R-R intervals (arrows). *N* = 3–4 mice for each WT and *Pkd2*-KO age-matched 6 months old.

Because heart conduction systems including SAN and AVN have an important role in electro-cardiac propagation (28, 29), electrocardiac properties were further analyzed in *Pkd2*-KO mice. The heart rate based on the *in vivo* electrocardiogram (ECG) was 583 ± 5 beats per minute for the WT mice and 625 ± 10 beats per minute for *Pkd2*-KO mice; we did not observe an apparent irregularity in ECG (Figure 4A). In order to evaluate the electrical activity of the heart in the absence of neurohumoral effects, we obtained an *ex vivo* ECG from isolated hearts (Figure 4B). Without neurohumoral effects, WT hearts showed a regular rhythm with 125 ± 20 beats per minute, and the *Pkd2*-KO hearts had arrhythmic heart rate of 127 ± 22 beats per minute evidence from atrial fibrillation or atrioventricular block characterized by irregular R-R intervals. Cardiac arrhythmia was also evidence in *Pkd2*-KO hearts at higher preloads.

Based on these analyses, our studies indicated that the *Pkd2*-KO hearts were hypertrophied with interstitial fibrosis and abnormal SAN and AVN. The impact of cardiac and conduction system fibrosis might thus result in arrhythmogenic hearts in *Pkd2*-KO mice.

### The *Pkd2*-KO Hearts Had Abnormal Systolic and Diastolic Functions

**Figure 5:**
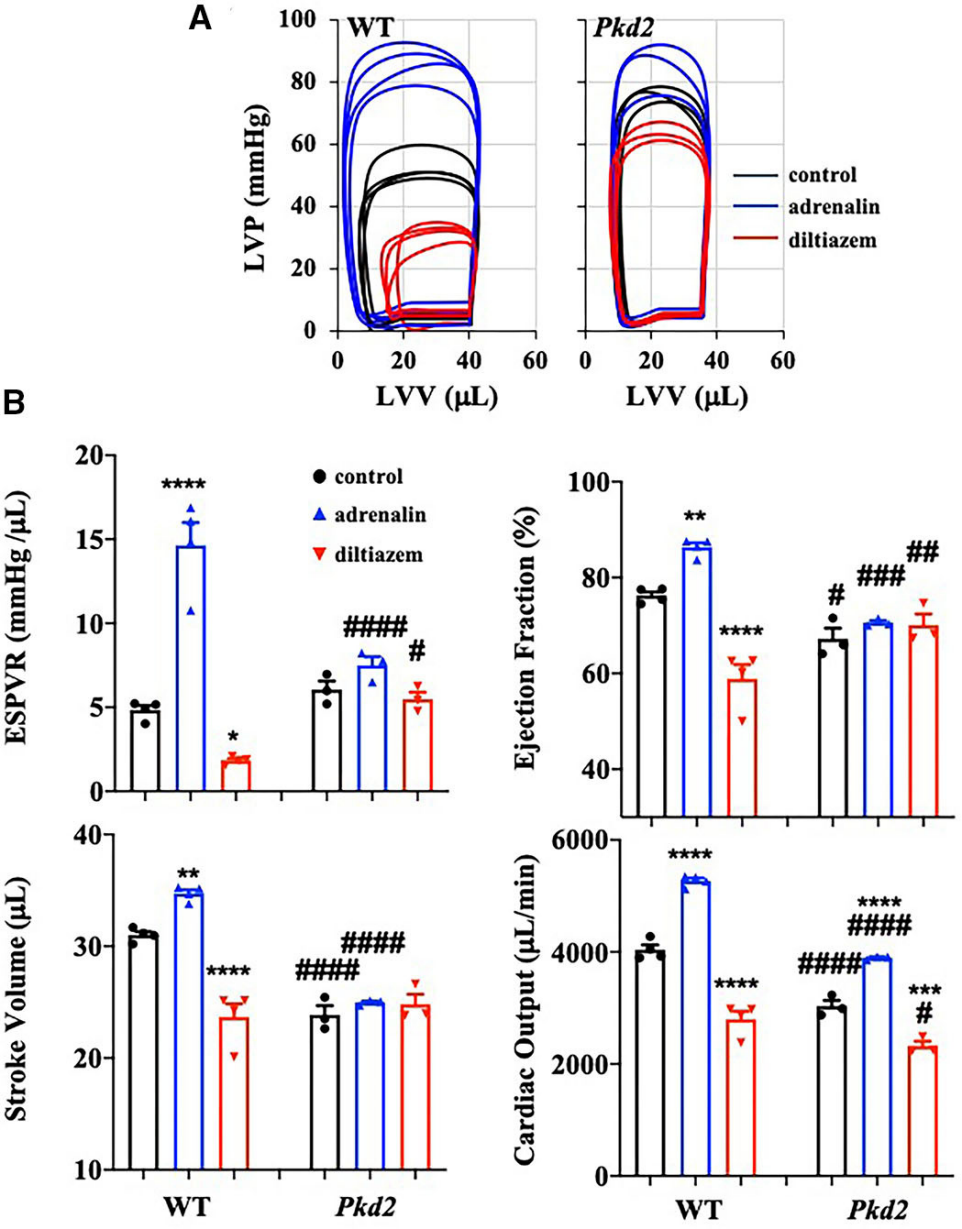
The cardiac function of *Pkd2*-KO mice is not responsive on changes in cardiac stresses. **(A)** Individual graphs of the relationship between left ventricle pressure (LVP) and left ventricle volume (LVV) heart function are shown before and after injection of adrenaline (4 mg/L) and diltiazem (0.08 mg/L) in wild-type (WT) and *Pkd2*-KO (Pkd2) hearts. **(B)** End-systolic-pressure-volume relationship (ESPVR, an index of systolic contractility function), stroke volume, ejection fraction, and cardiac output are used to evaluate the heart responses on positive-stress (adrenaline) and negative-stress (diltiazem). *N* = 3–4 mice for each WT and *Pkd2*-KO age-matched 6 months old. Statistical significance between control and treatment is indicated with asterisk (*) sign; between genotypes in hash (#). *p < 0.05; **p < 0.01; ***p < 0.001; ****p < 0.0001 compared to control within the same genotype. ^#^*p* < 0.05; ^*###*^*p* < 0.001; ^*####*^*p* < 0.0001 compared to the corresponding WT.

The heart-specific *Pkd2* mutant knockout did not show any behavior issue, at least not within the end-point of our studies at 6-months of age. However, because these mice had cardiac hypertrophy and fibrosis (Figure 1), heart functions were evaluated independently from the autonomic neuronal system by analyzing the left ventricular pressure-volume relationship (PV loop). PV loop allows a more precise analysis of heart functions by plotting the changes in left ventricular pressure and volume during each cardiac cycle. In order to quantify the changes in heart functions during physiologic stresses, LV parameters in both WT and *Pkd2*-KO were further evaluated in response to adrenaline (4 μg/L) or diltiazem (0.08 μg/L; Figure 5A). As expected, while adrenaline increased heart rate and contractility via β_1_-receptors, diltiazem had a negative inotropic and negative chronotropic effects in both WT and *Pkd2*-KO hearts (Supplementary Table 1). The end-systolic pressure volume relationship (ESPVR) is a relationship between LV pressure (LVP) and volume (LVV) at the end of the systole, and the ESPVR is considered a marker for LV systolic contractile function and elastance (30–32). Our results demonstrated that the LV ESPVR, stroke volume (SV), and ejection fraction (EF) didn't change significantly after adrenaline or diltiazem in *Pkd2*-KO hearts (Figure 5B), which could be a result of decreased heart muscle contractility due to fibrosis and systolic dysfunction of LV. Despite no changes in the ESPVR, SV, and EF in *Pkd2*-KO hearts, the cardiac output (CO) was significantly altered by adrenaline or diltiazem. Importantly, the CO in responses to the pharmacological agents was significantly less in *Pkd2*-KO than WT hearts primarily due to contractility dysfunction in *Pkd2*-KO hearts. The LV Pmax (maximum pressure) and LV ESP (end-systolic pressure) were higher in *Pkd2*-KO hearts before and after adrenaline and diltiazem, which could be due to LV fibrosis, stiffness, structural changes, and narrow LV chamber (Supplementary Table 1).

**Figure 6:**
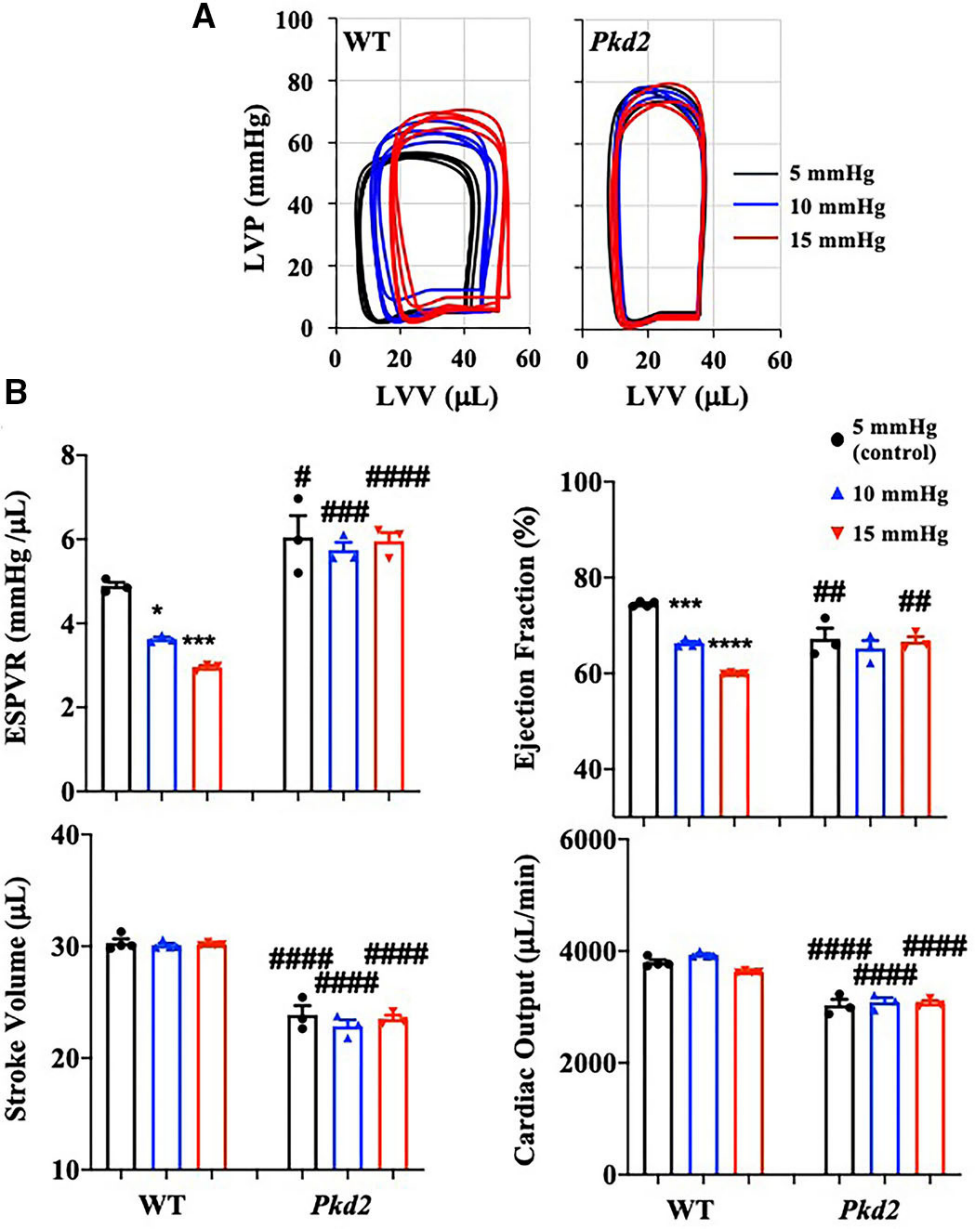
The cardiac function of *Pkd2*-KO mice is not responsive on changes in preloads. **(A)** Individual graphs of pressure-volume loop show effects of different preloads of 5, 10, and 15 mmHg on heart function. **(B)** End-systolic-pressure-volume relationship (ESPVR, an index of systolic contractility function), stroke volume, ejection fraction, and cardiac output are used to measure heart functions in response to different pre-load levels. *N* =3–4 mice for each WT and PKD age-matched 6 months old. Statistical significance between control and treatment is indicated with asterisk (*) sign; between genotypes in hash (#). *p < 0.05; ***p < 0.001; ****p < 0.0001; ^#^*p* < 0.05; ^*##*^*p* < 0.01; ^*###*^*p* < 0.001; ^*####*^*p* < 0.0001.

Preload is used to express EDV (end-diastolic volume); therefore, the higher the preload is, the greater the EDV is. EDV depends on left ventricular compliance during diastole. The 5, 10, and 15 mmHg preload pressures were therefore used to study EDV and other cardiac functions (Figure 6A). In *Pkd2*-KO mice, the EDV decreased and did not change with increasing preload due to abnormal LV expansion or compliance and filling impairment in the fibrotic hearts (Supplementary Table 2). Decreased EDV subsequently led to decrease in SV and CO significantly in *Pkd2*-KO compared to WT hearts (Figure 6B). Furthermore, dP/dt_min_ which is a valuable marker for diastolic function (25) was significantly decreased in *Pkd2*-KO than WT hearts, indicating *Pkd2*-KO hearts had a diastolic failure due to compliance and filling impairment. Finally, the ESPVR was significantly higher in *Pkd2*-KO than the WT hearts in all preloads (Figure 6B). The steep and leftward of ESPVR slope was most likely not due to the improved myocardial function, but it was due to a narrow chamber in the hypertrophic and fibrotic LV. In *Pkd2*-KO hearts, the EF could not compensate in response to different preloads due to abnormal contractility in the fibrotic LV. Higher preload did not affect *ex vivo* ECG in either rhythmic WT or arrhythmic *Pkd2*-KO hearts. The *ex vivo* heart rate was also not significantly altered by higher preloads in both mice (Figure 4B, Supplementary Table 1).

The *Pkd1* mutant myocytes have been previously shown to have abnormal contractile calcium (16, 33). To investigate if it was also the case in our *Pkd2* mutant, we used lentivirus shRNA knockdown system to examine the effects of *Pkd2* knockdown (KD) in contractility and calcium. The contractile efficacy in myocytes was calculated by measuring the changes in muscle displacement (cell shortening) for each myocyte beat. The infection and blocking efficiencies of different *Pkd2* shRNAs were first verified in a kidney cell line and were further examined in primary cultured myocytes (Supplementary Figure 1). The *Pkd2* shRNA-D was selected and used, because it showed the highest knockdown efficiency. The myocyte contractions and calcium oscillations in *Pkd2* knockdown myocytes occurred more frequently than in control myocytes (Supplementary Figure 2; Supplementary Videos 1, 2). Further, the intracellular contractile calcium levels from *Pkd2*-KD myocytes were significantly lower than those from the control. The *Pkd2*-KD myocytes consistently and significantly showed a decrease in contractility. We also confirmed the knockdown studies in cardiomyocytes using a mouse model with complete *Pkd2* knockout (Supplementary Figure 3; Supplementary Videos 3, 4). Mouse *Pkd2* knockout cardiomyocytes were characterized by increased contractile oscillation frequency, decreased contractile calcium and dampened contractile strength.

### Fibrotic Pathways Were Activated in Macrophage Infiltrated *Pkd2* Mutant Hearts

The contractile calcium and contractility were abnormal in single *Pkd2*-KO myocytes (Supplementary Figures 2, 3), and the ESPVR and SV among others were functionally impaired in *Pkd2*-KO hearts (Figures 4–6). Together with the arrhythmogenic characterization of *Pkd2*-KO hearts (Figure 4), we speculated that interstitial and SAN fibrosis together with hypertrophy and abnormal contractile calcium could play important roles in *Pkd2*-KO hearts, leading to impaired cardiac functions (Figures 1, 2).

**Figure 7:**
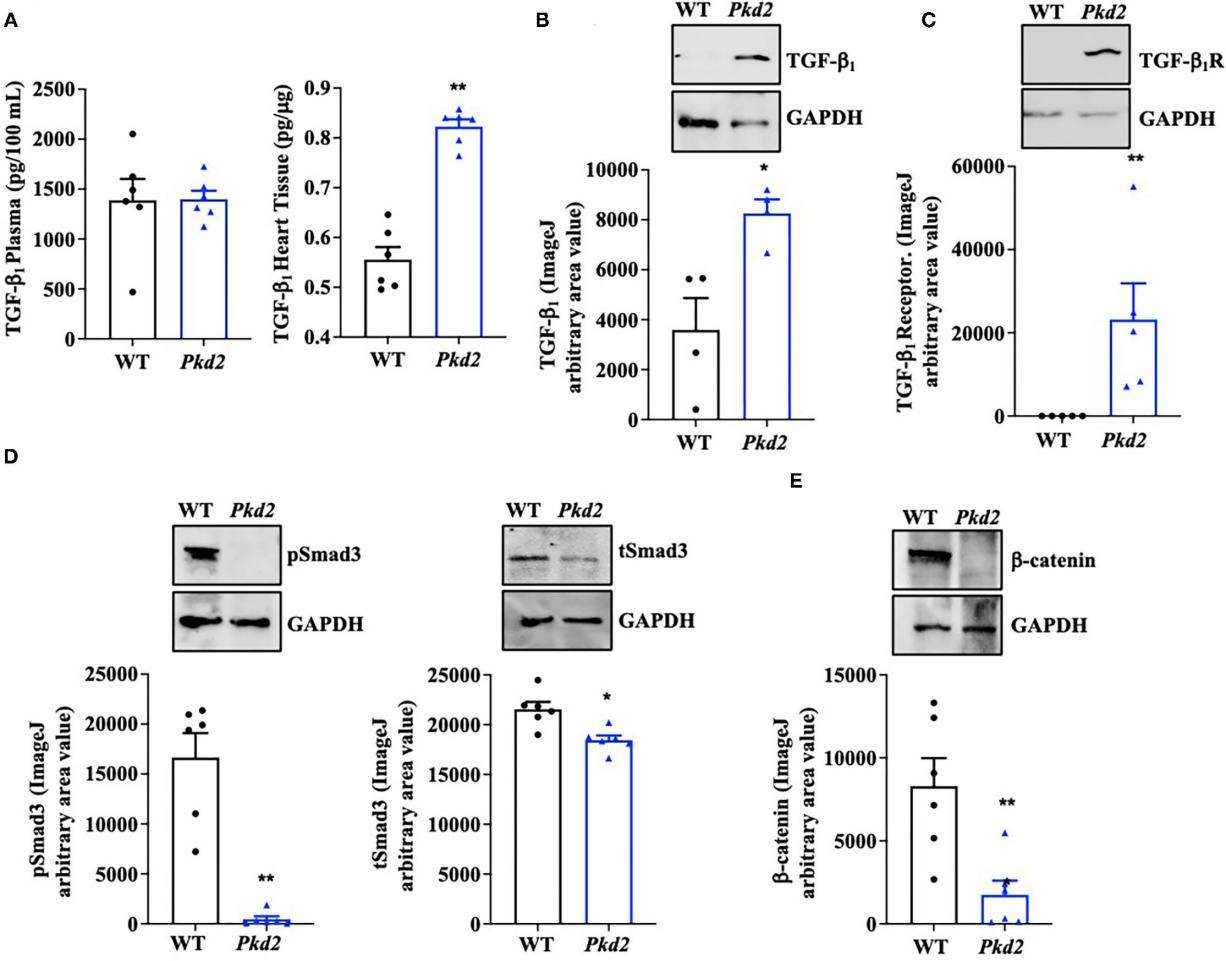
The hearts from *Pkd2*-KO mice demonstrate abnormal fibrotic pathways. **(A)** ELISA measurements of TGF-β_1_ were done in circulating plasma (left panel) and heart tissues (right panel). **(B,C)** Western blot analyses of mouse tissues show significantly higher expressions of TGF-β_1_ and TGF-β_1_ receptors in *Pkd2*-KO (*Pkd2*) compared to WT mice. **(D,E)** Western blot analyses of mouse tissues show significantly higher expressions of tSMAD3, pSmad3, and β-catenin in WT compared to *Pkd2*-KO mice. *N* = 4–6 mice for each WT and PKD age-matched 6 months old. *p < 0.05; **p < 0.01.

To examine if/how fibrosis pathways were activated, TGF-β_1_ expression in the circulating plasma and heart itself was analyzed with ELISA. While no difference was observed in the plasma level of TGF-β_1_, ELISA quantifications showed a significant increase of TGF-β_1_ in *Pkd2*-KO compared to WT hearts (Figure 7A). ELISA result was further supported by the Western blot data, which showed a significant increase in TGF-β_1_ expression level in *Pkd2*-KO compared to WT hearts (Figure 7B). We subsequently evaluated TGF-β_1_ receptor expression in the WT and *Pkd2*-KO hearts. The expression level of TGF-β_1_ receptor was significantly higher in *Pkd2*-KO than WT hearts (Figure 7C). To study the downstream effect of TGF-β_1_, we also evaluated phosphorylated-Smad3 (pSmad3), total-Smad3 (tSmad3), and β-catenin expression levels in the hearts. Our results showed higher expressions of pSmad3, tSmad3, and β-catenin in WT compared to *Pkd2*-KO hearts (Figures 7D,E, Supplementary Figure 4). While pSmad3 and tSmad3 were higher in WT hearts, the fractionation study showed that pSmad3 and tSmad3 were localized in nuclear fraction of *Pkd2*-KO myocytes (Supplementary Figure 5).

**Figure 8:**
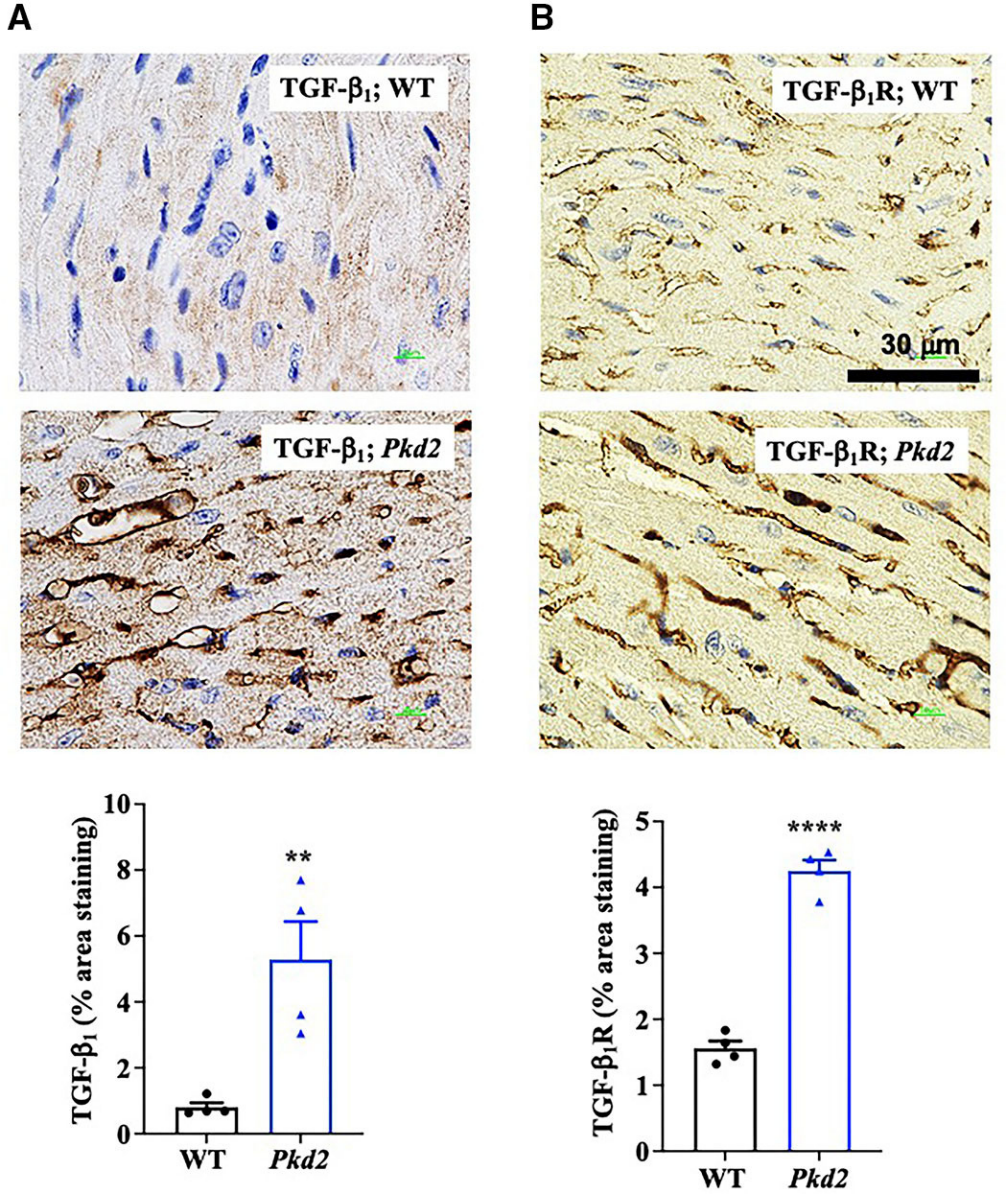
The hearts from *Pkd2*-KO mice are characterized with upregulated fibrotic pathways. **(A,B)** Immunohistochemistry staining demonstrated a significant increase of TGF-β_1_ and TGF-β_1_ receptor (dark brown color) in *Pkd2*-KO (*Pkd2*) hearts compared to the wild-type (WT). A total of 8 independent slides were used for each mouse. **p < 0.01; ****p < 0.0001.

**Figure 9:**
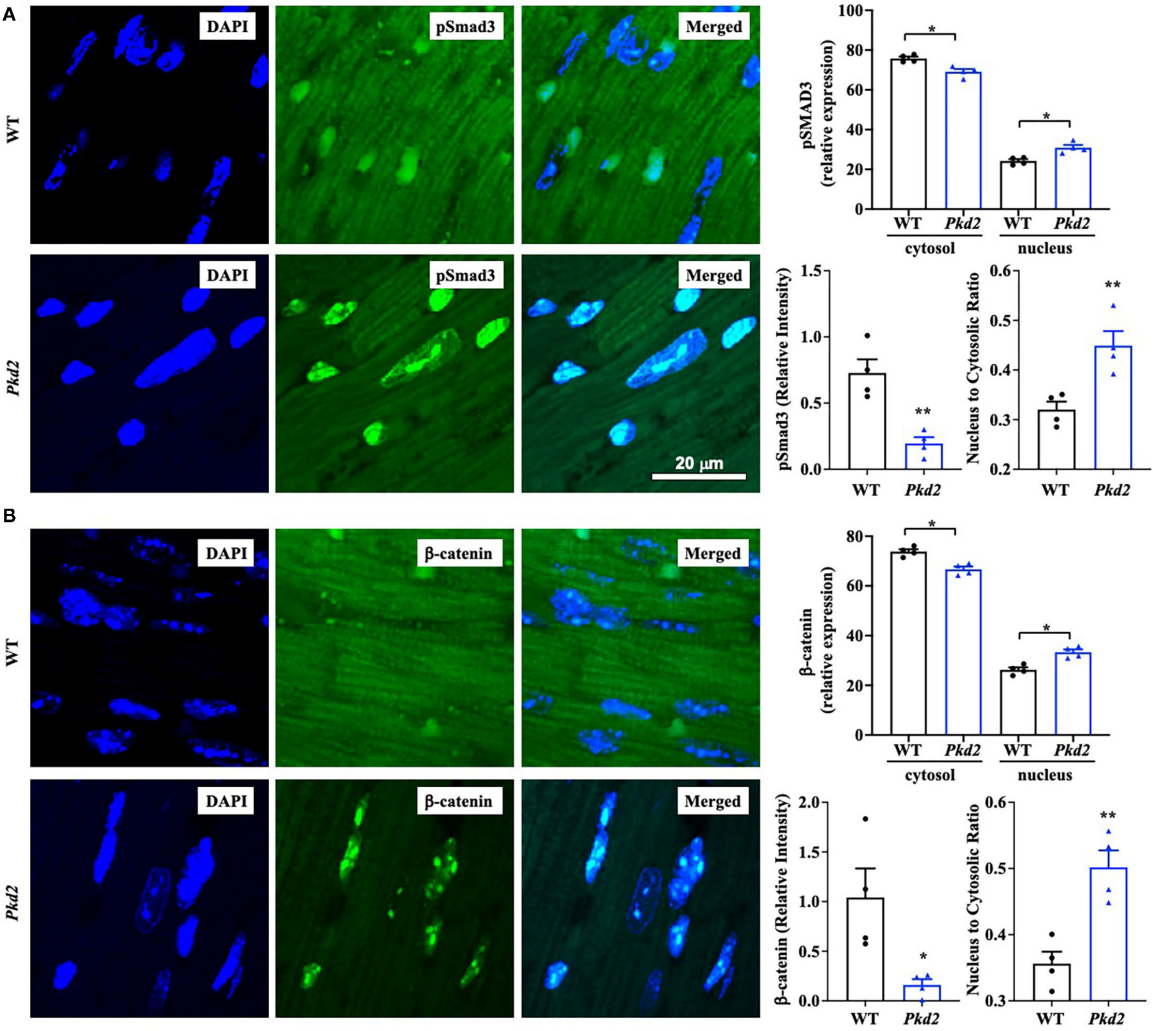
The hearts from *Pkd2*-KO mice are characterized with a significant increase in nuclear localization of pSMAD3 and β-catenin. **(A,B)** Immunofluorescence staining of pSmad3 **(A)** or β-catenin **(B)** revealed a significant increase in nuclear localization and nucleus to cytoplasm ratio of pSmad3 or β-catenin in *Pkd2*-KO (*Pkd2*) compared to wild-type (WT) hearts. *N* = 4 mice for each group. A total of 8 independent slides were used for each mouse. *p < 0.05; **p < 0.01.

To confirm the Western blot data, immunohistochemistry studies were performed to evaluate TGF-β_1_ and TGF-β_1_ receptor expressions. Consistent to our immunoblot results, expressions of both TGF-β_1_ and TGF-β_1_ receptors were significantly higher in *Pkd2*-KO compared to WT hearts (Figures 8A,B). The expression levels of pSmad3 and β-catenin were also analyzed with immunofluorescence studies. Consistent with our Western blot data, expressions of both pSmad3 and β-catenin in cytosol were significantly lower in *Pkd2*-KO compared to WT hearts (Figures 9A,B). The nucleus-to-cytosol ratio of both pSmad3 and β-catenin were significantly higher in *Pkd2*-KO than WT hearts, indicating greater the nuclear localizations of pSmad3 and β-catenin in *Pkd2*-KO hearts.

**Figure 10:**
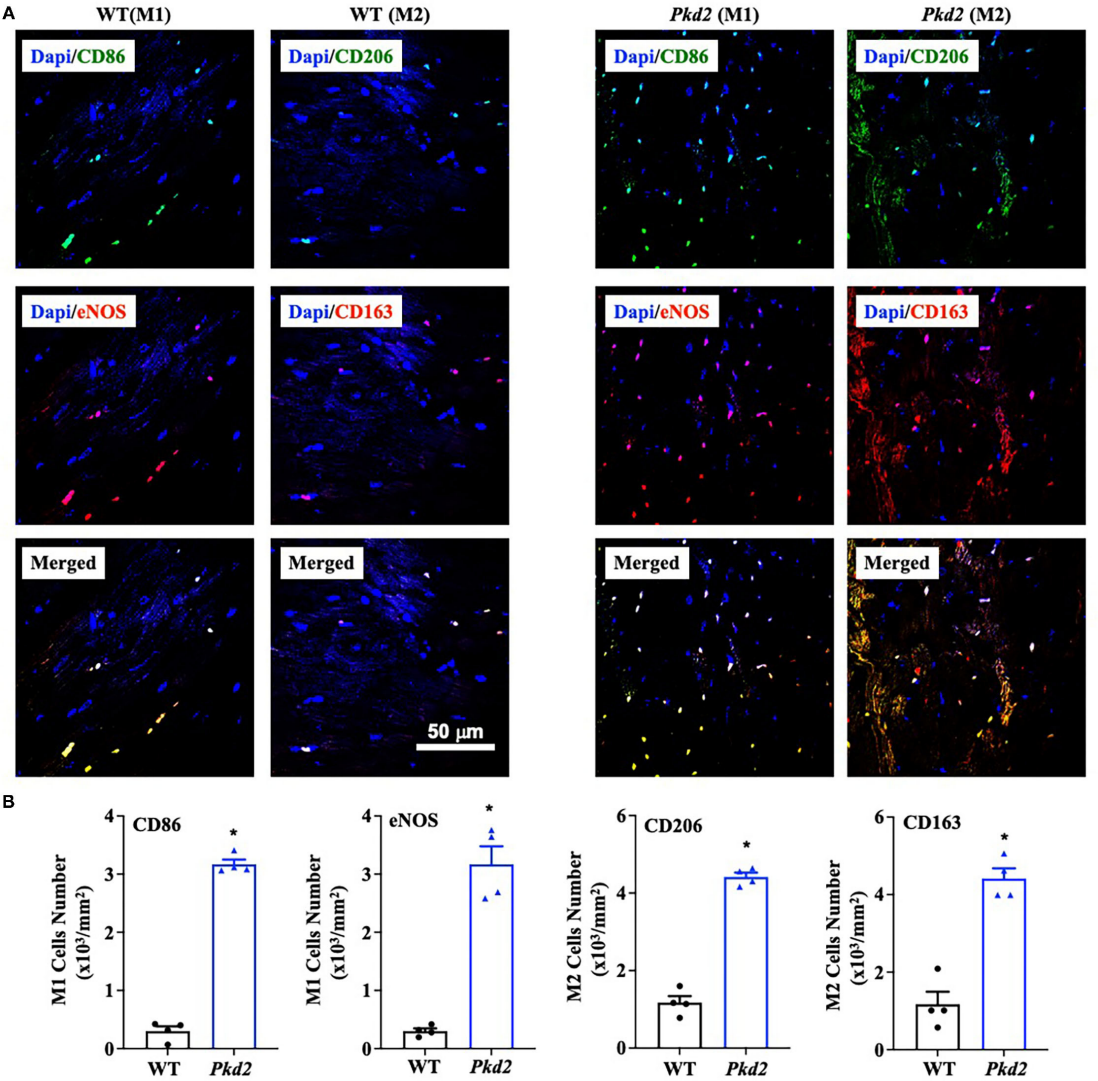
The hearts from *Pkd2*-KO mice are significantly infiltrated by macrophages. **(A,B)** Immunofluorescence staining revealed a significant higher number of macrophages M1 and M2. Image was quantified by binary masking of the red or green fluorescence image. *N* = 4 mice for each WT and *Pkd2*-KO age-matched 6 months old. *p < 0.05.

Because the *Pkd2*-KO hearts are characterized with a higher TGF-β_1_ expression, we next analyzed a potential inflammation in the *Pkd2*-KO hearts. We investigated potential infiltration in heart tissues by macrophage M1 and M2, which is known to be a local source for TGF-β_1_ (34). The immunofluorescence analyses revealed that macrophage M1 and M2 infiltration was significantly higher in the mouse *Pkd2*-KO compared to WT hearts (Figures 10A,B).

## Discussions

**Figure 11:**
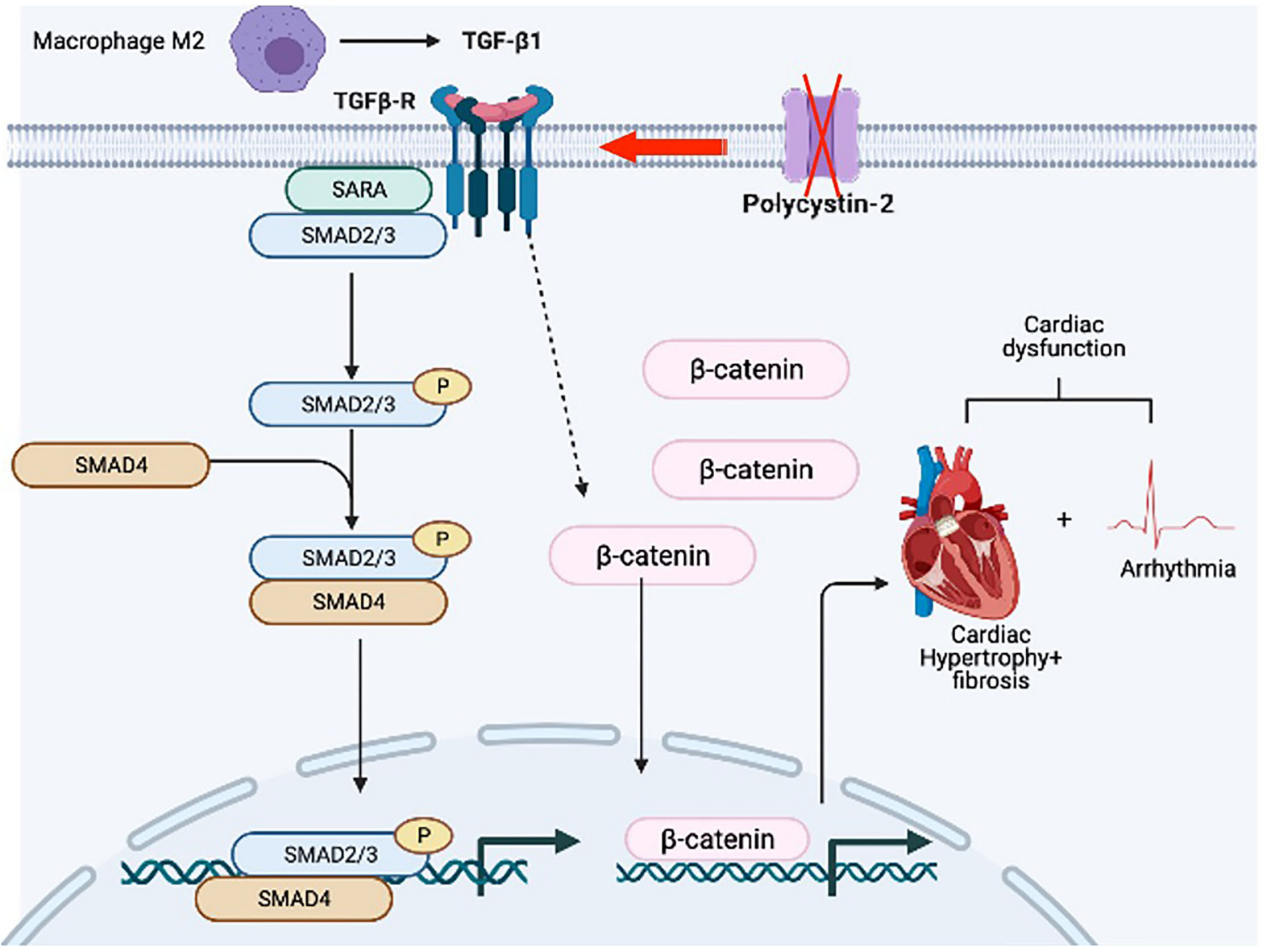
Hypothetical diagram summarizes the pathogeneses of PKD hearts. We hypothesize that hearts from *Pkd2*-KO mice are characterized by the overexpression of TGF-β_1_ receptor. Local TGF-β_1_ secretion by infiltrating macrophages further exacerbates the activation of fibrotic pathways resulting in hypertrophy and interstitial fibrosis. Subsequent activation of TGF-β_1_ receptor results in the activation of pSMAD3 and b-catenin in the myocytes. The fibrosis in the sinoatrial node and surrounding tissue can results in arrhythmogenic heart. All of which will cause systolic and diastolic cardiac dysfunction.

Cardiovascular complications remain a major cause of mortality and morbidity in patients with autosomal dominant polycystic kidney disease (PKD) (21), but the etiopathogenesis behind the complications is not completely understood. Our current research focused mainly on the impact of *Pkd2* gene mutation on heart tissues and functions. Based on the structural changes in the heart tissues, we showed clear evidence of cardiac remodeling, including LV hypertrophy, and interstitial fibrosis. The *Pkd2*-KO hearts showed a scarring process and cardiac muscle fibrosis with the fatty fibrotic changes in the sinoatrial node (SAN), which could serve as the underlying risk factor for cardiac arrhythmia in PKD patients. This tissue remodeling was thus associated with arrhythmogenic heart, systolic, and diastolic dysfunctions. Our studies also pointed to the involvement of macrophages, secreting anti-inflammatory TGF-β_1_, and TGF-β_1_ receptor expression in the *Pkd2*-KO heart tissues, and subsequently activating SMAD and β-catenin pathways in *Pkd2*-KO hearts (Figure 11).

A common event in all myocardial fibrotic remodeling involves either mechanical or chemical loading in the heart (35), although fibroblast cilia have also been recently shown to involve in the process (36). The chemical loading-induced fibrosis is usually associated with activation of immune cells and inflammation, leading to the increased extracellular matrix (36). Among the inflammatory cells, the macrophages play the most extensive role in secreting factors associated with inflammation. Our data showed that the macrophage M1 and M2 significantly infiltrated *Pkd2*-KO hearts. This suggested that M1 plays an essential role in cardiac inflammation, and it might play a role in the onset or progression of fibrosis in *Pkd2*-KO hearts (37). On the other hand, M2 may involve in matrix deposition and tissue remodeling in *Pkd2*-KO hearts (37). This suggested that our *Pkd2*-KO hearts were still undergoing the inflammation process and progressing toward fibrosis, although fibrosis and remodeling could be detected and confirmed in histochemistry analyses.

The roles of mediators TGF-β_1_, Smad, and β-catenin were next examined because they are generally known signaling pathways leading to cardiac fibrosis (35, 38–40). At least in the *Pkd2*-KO mouse model, circulating TGF-β_1_ did not seem to be increased, indicating that TGF-β_1_ might be restricted and localized within cardiac tissues. Of note is that not only TGF-β_1_ but also its receptor had increased in expressions and might have critical roles in the cardiac fibrosis. These studies were supported by immunoblotting, ELISA, and immunohistology studies. Both pSmad3 and β-catenin were significantly localized in the nucleus of *Pkd2*-KO myocytes, indicating a significant nuclear translocation and fibrotic signaling activation (38, 41). Immunofluorescent studies and immunoblots in mouse tissues indicated a lower total expression of pSmad3, tSMAD3, or β-catenin in *Pkd2*-KO hearts in cytosol. We further evaluated tSMAD3 and pSMAD3 expressions in the nucleus. Results indicated a higher nuclear localization of pSMAD3 in the *Pkd2*-KO compared to WT hearts. Because of the low cytosolic expression of pSmad3 or β-catenin in *Pkd2*-KO hearts, we further speculated that this might be a compensatory mechanism by the cardiac tissues to slow down the fibrotic process in *Pkd2*-KO tissues.

Given a strong pathogenesis association between cardiac fibrosis and myocardial dysfunction (42), we decided to study the heart functions using the *Pkd2*-KO mouse model (Supplementary Tables 1, 2). An isolated working heart system is commonly used to gather precise LV pressure-volume relations (PV loop) independent of systemic neuronal effect. PV loop is a powerful tool that provides us qualitative and quantitative measurements of the heart functions. PV loop provides many hemodynamic parameters that are not measurable by other methods, including elastance, contractility, and stroke work. The *Pkd2*-KO fibrotic hearts negatively affected expansion, compliance, and filling of LV as indicated by the end-diastolic volume (EDV). With less volume in the heart at the end of diastole (EDV), the stroke volume (SV) and cardiac output (CO) also decreased significantly. On the other hand, dP/dt_min_, a valuable marker for diastolic function, decreased in *Pkd2*-KO hearts. Based on these results, *Pkd2*-KO hearts were further confirmed to have diastolic failure due to compliance and filling impairment. It is known that oxygen extraction during diastole is higher than during systolic interval (43); perivascular fibrosis can therefore restrict the oxygen diffusion during diastole, which may further worsen diastolic function (43).

The end-systolic pressure-volume relationship (ESPVR) is considered a marker for left ventricular contractility and elastance (30–32). In *Pkd2*-KO hearts, the LV ESPVR did not change significantly after adrenaline and diltiazem injection due to fibrosis, decreased heart muscle contractility and systolic dysfunction of the LV. This was also supported by the results from the LV end-systolic volume (ESV), stroke volume (SV), and ejection fraction (EF). Moreover, the ESPVR was also analyzed in different preloads. The ESPVR with steep and leftward slope was significantly higher in *Pkd2*-KO hearts in all preloads, indicating a narrow chamber in the hypertrophic and fibrotic ventricle. In addition, the contractile calcium was significantly lower, resulting in weak contraction in *Pkd2* mutant myocytes (18).

Fibrotic tissue forms a barrier between cardiomyocytes, which can result not only in systolic dysfunction but also in electric conduction impairment (43). Increased fibrosis within and around SAN can cause structural destruction and dysfunction, leading to arrhythmia (44, 45). While no apparent arrhythmia was observed in the *Pkd2*-KO mouse model *in vivo*, the *Pkd2*-KO hearts were susceptible to arrhythmia when hearts were isolated away from neuronal input in the *ex vivo* studies. Atrial fibrillation (AF) or AV block was seen in isolated *Pkd2*-KO hearts. While the role of cardiac fibrosis is well-known in both atrial and ventricular arrhythmias, SAN dysfunction is a common finding in AF patients, particularly with advanced atrial fibrosis with SAN involvement (44). We also looked at HCN4, an ion channel responsible for the slow Na^+^ current in the heart conduction system. HCN-positive nodal cells were significantly decreased in the atria-ventricular node of *Pkd2*-KO hearts. Of note is that HCN4 channels are required for generating slow current, which is responsible for spontaneous depolarization of the specialized myocytes of the heart pacemaker (46).

In conclusion, our studies indicated fibrosis in *Pkd2*-KO hearts. Fibrosis in the conduction system might results in arrhythmia, and thus the PKD patients might be susceptible to atrial fibrillation (12) and potential arrhythmia-induced sudden cardiac death (47). Our studies implicated that cardiac fibrosis was also contributed to systolic and diastolic dysfunctions, which are seen in PKD patients (48). A potential prospect for therapeutic manipulation might therefore involve cardiac inflammation induced by TGF-b1.

## Materials and Methods

Throughout our studies, mice were used to examine the structural and functional changes of the heart. Our animal studies were approved by the Chapman University Institutional Animal Care and Use Committee (IACUC# 2020-1132 and PHS# D17-00960). The *Pkd2*^*flox*/*flox*^ mice were previously given from Dr. Jing Zhou's laboratory at Harvard Medical School (49, 50); these mice were bred with heart-specific transgenic mice *MyH6* (Jackson Laboratory; stock#011038). *MyH6*•*Pkd2*^*wt*/*wt*^ mouse is denoted as wild-type (WT; control), and *MyH6*•*Pkd*^*flox*/*flox*^ is designated as mutant or *Pkd2*-KO. Heart structure, function and electrocardiogram (ECG) were examined and measured at 6-months-old of age. Both male and female mice were used, and sex was identified to be an independent covariate to heart functions.

### Masson's Trichrome Staining

To evaluate myocadiac fibrosis and hypertrophy, heart tissues were collected and fixed in 10% formalin. The tissues were dehydrated in ethanol and xylene, embedded in liquid paraffin, and cut with a thickness of 5 μm. Cut sections were stained with Masson's trichrome kit (Cat# 25088-1; *Polysciences, Inc*.), and images were visualized and captured using KEYENCE BZ-X710 or Nikon Eclipse Ti microscope.

### Immunofluorescence Studies

To examine protein localization and expression, the formalin-fixed tissues were de-paraffinized. After deparaffinization and dehydration of paraffin sections, heat-induced epitope retrieval was performed using a pressure cooker and sodium citrate buffer (10 mM sodium citrate, 0.05% tween-20, pH 6.0). Once boiled, slides were transferred from phosphate-buffered saline (PBS) to the sodium citrate buffer in pressure cooker for 10 min, and then cooled to room temperature for 30 min. To permeabilize the tissues, slides were washed with permeabilization buffer containing 0.3% triton-100 in PBS for 10 min. After permeabilization, slides were blocked with 5% bovine serum albumin and 0.1% triton-100 in PBS solution and subsequently processed to detect specific proteins. All washing steps were done three times with PBS-T (tween-20, 0.05%).

For cell surface staining and cell size measurement, tissue slides were incubated with wheat germ agglutinin (WGA) (1:1,000; Cat# FL-1021; Vectorlabs) for 20 min. To detect specialized conduction cells in the atrioventricular node, tissues were incubated with rat anti-HCN4 antibody (1:1,000; Cat# ab32675; *Abcam, Inc*.) overnight at 4°C in a humidified chamber followed with AlexaFluor-488 goat anti-rat fluorescence secondary antibody (1:500; Cat# ab6840; *Abcam, Inc*). To study fibrotic pathways, anti-β-catenin (1:50; Cat# sc-133240; *Santa Cruz, Inc*.) and anti-pSMAD3 (1:1,000; Cat# sc-517575; *Santa Cruz, Inc*) antibodies were used in the same manner as anti-HCN4 antibody followed with AlexaFluor-488 secondary goat anti-mouse antibody (1:1,000; Cat# ab150113; *Abcam, Inc*). To evaluate macrophages infiltration, tissues were stained with anti-NOS2 antibody (1:50; Cat# sc-7271; *Santa Cruz, Inc*), and anti-CD-86 (1:50; Cat# sc-52448; *Santa Cruz, Inc*) for macrophages M1detection, anti-CD163 antibody (1:50; sc-20066; *Santa Cruz, Inc*), and anti-CD-206 (1:100; Cat# ab91992; *Signaling, Inc*) antibodies for macrophages M2 detection, followed with horse anti-mouse antibody Texas Red (1:1,000; Cat# NC9634446; *Thermo Fisher Scientific*), and AlexaFluor-488 goat anti-rat fluorescence secondary antibody (1:500; Cat# ab6840; *Abcam, Inc*), AlexaFluor-488 secondary goat anti-mouse antibody (1:1,000; Cat# ab150113; *Abcam, Inc*), and AlexaFluor-488 goat anti-rabbit fluorescence secondary antibody (1:500; Cat# ab91992; *Signaling, Inc*). In all cases, we used 4′,6-diamidino-2-phenylindole (DAPI) as a nuclear binding dye. All images were taken with the confocal A1R^+^ Nikon microscope (Version 4.30).

### Immunohistology

To examine protein localization and expression, the formalin-fixed tissues were de-paraffinized. After deparaffinization and dehydration of paraffin sections, heat-induced epitope retrieval was performed using a pressure cooker and sodium citrate buffer (10 mM sodium citrate, 0.05% tween-20, pH 6.0). Once boiled, slides were transferred from PBS to the sodium citrate buffer in pressure cooker for 10 min. Slides were then cooled to room temperature for 30 min, and permeabilized with permeabilization buffer containing 0.3% triton-100 in PBS for 10 min. To block endogenous peroxidase activity, slides were incubated in 3% hydrogen peroxide for 10 min and blocked with animal-free blocking solution (Cat# 15019; *Cell Signaling, Inc*.). After blocking, slides were incubated overnight at 4°C with mouse monoclonal anti-TGF-β_1_ (1:50; Cat# sc-130348; *Santa Cruz, Inc*.) or rabbit polyclonal anti-TGF-β_1_ receptor (1:100; Cat# ab235178; *Abcam, Inc*.) and subsequently incubated with SignalStain Boost Detection Reagent (HRP mouse; Cat# 8125; or HRP Rabbit; Cat# 8114; *Cell Signaling, Inc*.) for 30 min at room temperature. Slides were then incubated for 2–10 min with SignalStain DAB (Cat# 8059; *Cell Signaling, Inc*.), immersed in distilled H_2_O, stained with hematoxylin (Cat# 14166; *Cell Signaling, Inc*.) and mounted with coverslips. All washing steps were done three times with PBS-T (tween-20, 0.05%).

### Western Blot Analyses

To evaluate protein expressions, proteins were extracted from the tissues using radioimmunoprecipitation assay (RIPA) lysis buffer (Cat# 89901, *Thermo Fisher, Inc*.) containing Halt protease inhibitor cocktail (Cat# 78425, *Thermo Fisher, Inc*.). Total protein was quantified using the Bradford assay. Extracted proteins (25–50 μg) were loaded into 10% sodium dodecyl sulfate-polyacrylamide gel electrophoresis (SDS-PAGE) gel and transferred onto nitrocellulose membrane afterward. For nuclear fractionation, immunoprecipitation with anti-Histone H3 was used and loaded to the gels. The membrane was then blocked with 5% dry milk and subsequently processed to detect specific proteins using a standard method.

We used the following primary antibodies: rabbit polyclonal anti-TGF-β_1_ (1:1,000; Cat# ab92486; *Abcam, Inc*.), rabbit polyclonal anti-TGF-β_1_ receptor (1:1,000; Cat# ab31013; *Abcam, Inc*.), mouse monoclonal anti-β-catenin (1:100; Cat# sc-133240; *Santa Cruz, Inc*.), rabbit monoclonal pSmad3 (1:1,000; Cat# 9520s; *Cell Signaling, Inc*.), rabbit monoclonal Smad3 (1:1,000; Cat# 9523s; *Cell Signaling, Inc*.), rabbit polyclonal histone H3 (1:1,000; Cat# 9715s; *Cell Signaling, Inc*.), and anti-GAPDH (1:100; Cat# sc-365062; *Santa Cruz, Inc*.). Except for anti-GAPDH antibody which was already tagged with horseradish peroxidase (HRP), HRP-tagged secondary antibodies were used accordingly, followed with chemiluminescent substrate (Cat# 34577; *Thermo Fisher Scientific, Inc*.). Protein detection was carried out with Bio-Rad imager (ChemiDoc™ XRS+ System with Image Lab™ Software; Cat# 1708265; *Bio-Rad, Inc*.).

### ShRNA Knockdown

Lentiviral vectors containing shRNA to *Pkd2* knockdown (*Origene*; pGFP-C-shLenti clone ID: TL310397) were transfected into HEK293T cells. Viral supernatants were collected and pooled at 24- and 48-h post-transfection. Primary mouse myocytes were then incubated with pseudoviral particles and 8 μg/ml polybrene for 48 h prior to analysis. Transduction efficiency was observed via GFP reporter fluorescence, and *Pkd2* knockdown was verified through Western blot analysis. The shRNA sequences used in our experiments are shown below.

**Table:** 

**Descriptions**	**Sequences**
scrambled control	5′-TGA CCA CCC TGA CCT ACG GCG TGC AGT GC-3′
*Pkd2* A	5′-TTG TGC ATC TTG ACC TAC GGC ATG ATG AG-3′
*Pkd2* B	5′-TAC GGC ATG ATG AGC TCC AAT GTG TAC TA-3′
*Pkd2* C	5′-TTT GAT TTC TTC CTG GCA GCC TGT GAG AT-3′
*Pkd2* D	5′-GTC TGG ATT AAG CTC TTC AAA TTC ATC AA-3′

### Cytosolic Calcium Measurements

To observe cytosolic calcium in cultured cardiomyocytes, cells were incubated with 10 μM fura-2AM (Invitrogen) and protected from light for 30 min. Calcium was measured by comparing fura-2 excitation at 340 nm (bound calcium) and 380 nm (free calcium). After each experiment, free cytosolic calcium was determined by perfusing ionomycin (10 μM final concentration) to obtain the maximum calcium signal followed by the addition of EGTA (2 mM final concentration) to observe the minimum calcium signal. All experiments were performed at 37°C in a heated microscope chamber.

### Enzyme-Linked Immunosorbent Assay (ELISA)

To detect TGF-β_1_ in the circulating plasma, blood was collected from a submandibular vein (cheek punch) using a heparinized tube. Within 30 min of collection, blood was centrifuged for 15 min at 1000 × g at 4 °C, and supernatant was collected for the ELISA assay. To detect TGF-β_1_ in the heart, the heart tissues were collected and homogenized in RIPA buffer containing Halt protease inhibitor cocktail, and total protein was quantified with Bradford assay from a commercially available kit (Cat# PI23227; *ThermoFisher Scientific, Inc*.).

The TGF-β_1_ level was measured using a commercially available mouse TGF-β_1_ ELISA kit (Cat# LS-F5184, *LSBio, Inc*.). The assay was based on the sandwich ELISA method. Each well had been pre-coated with TGF-β_1_ antibody. Tissue lysate samples were added to the wells, and the target antigen (TGF-β_1_) was bound to the antibody. The unbounded proteins were washed away, and biotin-conjugated detection antibody was added, which bound to the captured antigen. Avidin-tagged HRP conjugate was added to bind with biotin. The TMB (3,3′,5,5′-tetramethylbenzidine) detection substrate was added to react with the HRP enzyme leading to color development, which was proportional to total TGF-β1 bound. A stop solution (sulfuric acid) was added to terminate the color development reaction, and the optical density of each well was measured at a wavelength of 450 nm.

### Electrocardiogram (ECG) and Heart Functions

To study isolated heart functions, the 6-month-old mouse was injected with heparin (100 units; IP) to prevent blood coagulation and anesthetized with ketamine 8–10 min later (200-350 mg/kg; IP). Mouse's chest was wiped clean with water, dried, and shaved to obtain *in vivo* ECG. The ultrasonic gel was warmed to 37°C to reduce stress to the mouse. The electrodes were then placed onto the mouse's chest. One electrode was placed around the xiphoid process of the left side of (the) sternum, and the other electrode at the 4th or 5th intercostal space on the right side. The ECG reading was taken at 5-s interval.

Immediately after obtaining *in vivo* ECG, an incision was made in the mid-abdomen toward the diaphragm. The diaphragm was opened, the thoracic cage was cut bilaterally, and the heart was dissected out. Immediately after the heart dissection, the aorta was cannulated and perfused with Krebs-Ringer solution (125 mM NaCl, 2.5 mM KCl, 1.25 mM NaH2PO4, 2 mM CaCl2, 1 mM MgCl_2_, 25 mM NaHCO_3_, and 25 mM glucose), which was continuously bubbled with carbogen (95% O_2_ and 5% CO_2_) to reach a pH of 7.4 at 38.0°C.

The *ex-vivo* perfusion of the mouse's heart was performed, the left atrium was cannulated, and cardiac function parameters were recorded using the working heart system (Supplementary Video 5; *Emka Technology, Inc*.). This approach provided quantitative measurements and heart functions that other methods cannot measure, including elastance, contractility, and stroke work. For evaluation of cardiac electrical activity in the absence of neurohumoral factors, an *ex vivo* ECG was obtained from the software after placing ECG electrodes on the right atrium and apex of the heart. Other cardiac parameters, including left ventricle pressure (LVP), left atrial pressure (LAP), aortic pressure (AP), and aortic flow (AoF) were obtained from the software. The preload and afterload were adjusted manually as needed.

The heart's responses to the external stresses were examined with a pharmacological stress test. The stress test was performed by using epinephrine (4 μg/L) or diltiazem (0.08 μg/L). E*nd-systolic pressure-volume relationship* (ESPVR), left ventricle pressure maximum (LVP max), and end-systolic pressure (ESP), left ventricle end-systolic volume (LV ESV), stroke volume (SV), and ejection fraction (EF) were obtained from the software or pressure-volume analysis. In some cases, preload was adjusted from 5 mmHg to either 10 or 15 mmHg. We used preload to indicate end-diastolic volume (EDV); therefore, the higher the preload was, the greater the EDV was. Preload was changed manually on the preload reservoir graduated cylinder, located on the isolated working heart system. After increasing the preload, LV pressure max (LV Pmax), end-systolic pressure (ESP), end-diastolic pressure (EDP), end-systolic volume (ESV), end-diastolic volume (EDV), stroke volume (SV), and ejection fraction (EF) were obtained from the software data and pressure-volume analysis.

### Data and Statistical Analysis

Image analyses were performed in Nikon NIS-Element for Advanced Research software (Version 4.51). This software was also used for image capture, segmentation, 3D object reconstruction, and automatic object recognition. A Photometric Coolsnap EZ CCD Monochrome Digital Camera was used to capture images with a 1392 × 1040 imaging array to resolve fine details of the images. Binary masking was used to calculate cell size, fibrosis, ventricular thickness, and image intensity for protein expression and localization based on granularity, shape, size, and pixel intensity. Quantitation of Western blots was done with the NIH Fiji ImageJ (Version 2.1). After a box of the intended proteins was drawn using the “gel” analysis function, the area under the curve was measured for the intensity of each protein band. All images were finalized on a six-core Mac Pro, 3.9 GHz to facilitate complete data extraction. Scale bars are provided in all figures and videos to indicate the actual image size.

A list of heart functions was measured or calculated using *Emka Technology* software. The software captured and recorded real-time data for electrical heart propagation, cardiac contractility, heart rate, left ventricle pressure and volume, preload, afterload, aortic pressure, and aortic outflow. These data were further used to calculate stroke volume, cardiac output, end-diastolic/systolic volume, and left atrial pressure rise and fall. Additional analyses were performed in Microsoft Excel for macOS (Version 16.48).

Our statistical analyses were performed using GraphPad Prism for macOS (Version 9.1). To compare two groups of unpaired datasets, a non-parametric student *t*-test was used with two-tail assuming no Gaussian distribution. For comparison within 3 or more groups with no matching datasets, we used non-parametric or mixed ordinary one-way ANOVA. The mean of each group was then compared with the mean of every other group using Tukey *post-hoc* multiple comparison test with multiplicity adjusted *P*-value for each comparison. *P* < 0.05 was considered significant in our studies. A more precise *P*-value was reported separately in the graphs. The total measurements and N values were also reported independently in each figure legend.

## Data Availability Statement

The original contributions presented in the study are included in the article/Supplementary Materials, further inquiries can be directed to the corresponding author.

## Ethics Statement

The animal study was reviewed and approved by Chapman University IACUC.

## Author Contributions

FA collected, analyzed data, drafted the manuscript, and oversaw the experimental progress. RP bred mice and served as a double-blind operator. KS assisted in tissue processing, working heart system, and served as another double-blind operator. BM performed calcium imaging studies. SN drafted the manuscript, designed research, and oversaw the study. All authors were participating in finalizing the manuscript.

## Funding

This work was supported in part by the NIH HL147311 and AHA 19IPLOI34730020. Denisse Larin-Henriquez and Maki Takahashi assisted FA, RP, and KS in reagent preparation and chemical ordering at Chapman University.

## Conflict of Interest

The authors declare that the research was conducted in the absence of any commercial or financial relationships that could be construed as a potential conflict of interest.

## Publisher's Note

All claims expressed in this article are solely those of the authors and do not necessarily represent those of their affiliated organizations, or those of the publisher, the editors and the reviewers. Any product that may be evaluated in this article, or claim that may be made by its manufacturer, is not guaranteed or endorsed by the publisher.

## References

[1] Zhang Y Dai Y Raman A Daniel E Metcalf J Reif G. Overexpression of TGF-beta1 induces renal fibrosis and accelerates the decline in kidney function in polycystic kidney disease. Am J Physiol Renal Physiol. (2020) 319:F1135–48. 10.1152/ajprenal.00366.2020 33166182 PMC7792699

[2] Norman J. Fibrosis and progression of autosomal dominant polycystic kidney disease (ADPKD). Biochim Biophys Acta. (2011) 1812:1327–36. 10.1016/j.bbadis.2011.06.012 21745567 PMC3166379

[3] Ratnam S Nauli SM. Hypertension in autosomal dominant polycystic kidney disease: a clinical and basic science perspective. Int J Nephrol Urol. (2010) 2:294−308. 25364490 PMC4215423

[4] Menezes LF Germino GG. The pathobiology of polycystic kidney disease from a metabolic viewpoint. Nat Rev Nephrol. (2019) 15:735–49. 10.1038/s41581-019-0183-y 31488901

[5] Torres VE Harris PC. Progress in the understanding of polycystic kidney disease. Nat Rev Nephrol. (2019) 15:70–2. 10.1038/s41581-018-0108-1 30607031 PMC6543819

[6] Bergmann C Guay-Woodford LM Harris PC Horie S Peters DJM Torres VE. Polycystic kidney disease. Nat Rev Dis Primers. (2018) 4:50. 10.1038/s41572-018-0047-y 30523303 PMC6592047

[7] Chapman AB Johnson AM Rainguet S Hossack K Gabow P Schrier RW. Left ventricular hypertrophy in autosomal dominant polycystic kidney disease. J Am Soc Nephrol. (1997) 8:1292–7. 10.1681/ASN.V881292 9259356

[8] Gabow PA Johnson AM Kaehny WD Kimberling WJ Lezotte DC Duley IT. Factors affecting the progression of renal disease in autosomal-dominant polycystic kidney disease. Kidney Int. (1992) 41:1311–9. 10.1038/ki.1992.195 1614046

[9] Suwa Y Higo S Nakamoto K Sera F Kunimatsu S Masumura Y. Old-Age onset progressive cardiac contractile dysfunction in a patient with polycystic kidney disease harboring a PKD1 frameshift mutation. Int Heart J. (2019) 60:220–5. 10.1536/ihj.18-184 30464138

[10] Leier CV Baker PB Kilman JW Wooley CF. Cardiovascular abnormalities associated with adult polycystic kidney disease. Ann Intern Med. (1984) 100:683–8. 10.7326/0003-4819-100-5-683 6231874

[11] Pirson Y Chauveau D Torres V. Management of cerebral aneurysms in autosomal dominant polycystic kidney disease. J Am Soc Nephrol. (2002) 13:269–76. 10.1681/ASN.V131269 11752048

[12] Yu TM Chuang YW Yu MC Huang ST Chou CY Lin CL. New-onset atrial fibrillation is associated with polycystic kidney disease: a nationwide population-based Cohort Study. Medicine. (2016) 95:e2623. 10.1097/MD.0000000000002623 26825919 PMC5291589

[13] Romao EA Moyses Neto M Teixeira SR Muglia VF Vieira-Neto OM Dantas M. Renal and extrarenal manifestations of autosomal dominant polycystic kidney disease. Braz J Med Biol Res. (2006) 39:533–8. 10.1590/S0100-879X2006000400014 16612477

[14] Chapman AB Stepniakowski K Rahbari-Oskoui F. Hypertension in autosomal dominant polycystic kidney disease. Adv Chronic Kidney Dis. (2010) 17:153–63. 10.1053/j.ackd.2010.01.001 20219618 PMC2845913

[15] Sharif-Naeini R Folgering JH Bichet D Duprat F Lauritzen I Arhatte M. Polycystin-1 and−2 dosage regulates pressure sensing. Cell. (2009) 139:587–96. 10.1016/j.cell.2009.08.045 19879844

[16] Altamirano F Schiattarella GG French KM Kim SY Engelberger F Kyrychenko S. Polycystin-1 assembles with Kv channels to govern cardiomyocyte repolarization and contractility. Circulation. (2019) 140:921–36. 10.1161/CIRCULATIONAHA.118.034731 31220931 PMC6733647

[17] Criollo A Altamirano F Pedrozo Z Schiattarella GG Li DL Rivera-Mejias P. Polycystin-2-dependent control of cardiomyocyte autophagy. J Mol Cell Cardiol. (2018) 118:110–21. 10.1016/j.yjmcc.2018.03.002 29518398

[18] Pedrozo Z Criollo A Battiprolu PK Morales CR Contreras-Ferrat A Fernandez C. Polycystin-1 is a cardiomyocyte mechanosensor that governs L-type Ca2+ channel protein stability. Circulation. (2015) 131:2131–42. 10.1161/CIRCULATIONAHA.114.013537 25888683 PMC4470854

[19] Anyatonwu GI Estrada M Tian X Somlo S Ehrlich BE. Regulation of ryanodine receptor-dependent calcium signaling by polycystin-2. Proc Natl Acad Sci USA. (2007) 104:6454–9. 10.1073/pnas.0610324104 17404231 PMC1851053

[20] Morita H. Secondary cardiomyopathy in polycystic kidney disease syndrome. Int Heart J. (2019) 60:10–11. 10.1536/ihj.18-514 30686801

[21] Fick GM Johnson AM Hammond WS Gabow PA. Causes of death in autosomal dominant polycystic kidney disease. J Am Soc Nephrol. (1995) 5:2048–56. 10.1681/ASN.V5122048 7579053

[22] Kang YR Ahn JH Kim KH Choi YM Choi J Park JR. Multiple cardiovascular manifestations in a patient with autosomal dominant polycystic kidney disease. J Cardiovasc Ultrasound. (2014) 22:144–7. 10.4250/jcu.2014.22.3.144 25309692 PMC4192413

[23] Bardaji A Vea AM Gutierrez C Ridao C Richart C Oliver JA. Left ventricular mass and diastolic function in normotensive young adults with autosomal dominant polycystic kidney disease. Am J Kidney Dis. (1998) 32:970–5. 10.1016/S0272-6386(98)70071-X 9856512

[24] de Chickera S Akbari A Levin A Tang M Brown P Djurdev O. The risk of adverse events in patients with polycystic kidney disease with advanced chronic kidney disease. Can J Kidney Health Dis. (2018) 5:2054358118774537. 10.1177/2054358118774537 30186614 PMC6117870

[25] Schrier RW. Renal volume, renin-angiotensin-aldosterone system, hypertension, and left ventricular hypertrophy in patients with autosomal dominant polycystic kidney disease. J Am Soc Nephrol. (2009) 20:1888–93. 10.1681/ASN.2008080882 19696226

[26] Saito M Okayama H Yoshii T Higashi H Morioka H Hiasa G. Clinical significance of global two-dimensional strain as a surrogate parameter of myocardial fibrosis and cardiac events in patients with hypertrophic cardiomyopathy. Eur Heart J Cardiovasc Imaging. (2012) 13:617–23. 10.1093/ejechocard/jer318 22271116

[27] Travers JG Kamal FA Robbins J Yutzey KE Blaxall BC. Cardiac fibrosis: the fibroblast awakens. Circ Res. (2016) 118:1021–40. 10.1161/CIRCRESAHA.115.306565 26987915 PMC4800485

[28] Bartos DC Grandi E Ripplinger CM. Ion channels in the heart. Compr Physiol. (2015) 5:1423–64. 10.1002/cphy.c140069 26140724 PMC4516287

[29] Moorman AF de Jong F Denyn MM Lamers WH. Development of the cardiac conduction system. Circ Res. (1998) 82:629–44. 10.1161/01.RES.82.6.629 9546372

[30] Walley KR. Left ventricular function: time-varying elastance and left ventricular aortic coupling. Crit Care. (2016) 20:270. 10.1186/s13054-016-1439-6 27613430 PMC5018161

[31] Aghajani E Muller S Kjorstad KE Korvald C Nordhaug D Revhaugand A. The pressure-volume loop revisited: is the search for a cardiac contractility index a futile cycle? Shock. (2006) 25:370–6. 10.1097/01.shk.0000209521.20496.7a 16670639

[32] Kass DA Beyar R Lankford E Heard M Maughan WL Sagawa K. Influence of contractile state on curvilinearity of in situ end-systolic pressure-volume relations. Circulation. (1989) 79:167–78. 10.1161/01.CIR.79.1.167 2910541

[33] Lee JJ Cheng SJ Huang CY Chen CY Feng L Hwang DY. Primary cardiac manifestation of autosomal dominant polycystic kidney disease revealed by patient induced pluripotent stem cell-derived cardiomyocytes. EBioMedicine. (2019) 40:675–84. 10.1016/j.ebiom.2019.01.011 30639418 PMC6413318

[34] Wang Y Zhang L Wu GR Zhou Q Yue H Rao LZ. MBD2 serves as a viable target against pulmonary fibrosis by inhibiting macrophage M2 program. Sci Adv. (2021) 7:eabb6075. 10.1126/sciadv.abb6075 33277324 PMC7775789

[35] Yan W Wang P Zhao CX Tang J Xiao X Wang DW. Decorin gene delivery inhibits cardiac fibrosis in spontaneously hypertensive rats by modulation of transforming growth factor-beta/Smad and p38 mitogen-activated protein kinase signaling pathways. Hum Gene Ther. (2009) 20:1190–200. 10.1089/hum.2008.204 19697998

[36] Villalobos E Criollo A Schiattarella GG Altamirano F French KM May HI. Fibroblast primary cilia are required for cardiac fibrosis. Circulation. (2019) 139:2342–57. 10.1161/CIRCULATIONAHA.117.028752 30818997 PMC6517085

[37] Atri C Guerfali FZ Laouini D. Role of human macrophage polarization in inflammation during infectious diseases. Int J Mol Sci. (2018) 19:1801. 10.3390/ijms19061801 29921749 PMC6032107

[38] Xiang FL Fang M Yutzey KE. Loss of beta-catenin in resident cardiac fibroblasts attenuates fibrosis induced by pressure overload in mice. Nat Commun. (2017) 8:712. 10.1038/s41467-017-00840-w 28959037 PMC5620049

[39] Ma F Li Y Jia L Han Y Cheng J Li H. Macrophage-stimulated cardiac fibroblast production of IL-6 is essential for TGF beta/Smad activation and cardiac fibrosis induced by angiotensin II PLoS ONE. (2012) 7:e35144. 10.1371/journal.pone.0035144 22574112 PMC3344835

[40] Attaran S Sherwood R Dastidar MG El-Gamel A. Identification of low circulatory transforming growth factor beta-1 in patients with degenerative heart valve disease. Interact Cardiovasc Thorac Surg. (2010) 11:791–3. 10.1510/icvts.2010.244384 20736227

[41] Venkatesan N Pini L Ludwig MS. Changes in Smad expression and subcellular localization in bleomycin-induced pulmonary fibrosis. Am J Physiol Lung Cell Mol Physiol. (2004) 287:L1342–7. 10.1152/ajplung.00035.2004 15333293

[42] Webber M Jackson SP Moon JC Captur G. Myocardial fibrosis in heart failure: anti-fibrotic therapies and the role of cardiovascular magnetic resonance in drug trials. Cardiol Ther. (2020) 9:363–76. 10.1007/s40119-020-00199-y 32862327 PMC7584719

[43] Piek A de Boer RA Sillje HH. The fibrosis-cell death axis in heart failure. Heart Fail Rev. (2016) 21:199–211. 10.1007/s10741-016-9536-9 26883434 PMC4762920

[44] Csepe TA Kalyanasundaram A Hansen BJ Zhao J Fedorov VV. Fibrosis: a structural modulator of sinoatrial node physiology and dysfunction. Front Physiol. (2015) 6:37. 10.3389/fphys.2015.00037 25729366 PMC4325882

[45] Akoum N McGann C Vergara G Badger T Ranjan R Mahnkopf C. Atrial fibrosis quantified using late gadolinium enhancement MRI is associated with sinus node dysfunction requiring pacemaker implant. J Cardiovasc Electrophysiol. (2012) 23:44–50. 10.1111/j.1540-8167.2011.02140.x 21806700 PMC4465539

[46] DiFrancesco D. The role of the funny current in pacemaker activity. Circ Res. (2010) 106:434–46. 10.1161/CIRCRESAHA.109.208041 20167941

[47] Yang B Wang Q Wang R Xu T. Clinical manifestation, management and prognosis of acute myocardial infarction in autosomal dominant polycystic kidney disease. Kidney Blood Press Res. (2018) 43:1806–12. 10.1159/000495638 30504716

[48] Huebener P Abou-Khamis T Zymek P Bujak M Ying X Chatila K. CD44 is critically involved in infarct healing by regulating the inflammatory and fibrotic response. J Immunol. (2008) 180:2625–33. 10.4049/jimmunol.180.4.2625 18250474

[49] Pala R Mohieldin AM Sherpa RT Kathem SH Shamloo K Luan Z. Ciliotherapy: remote control of primary cilia movement and function by magnetic nanoparticles. ACS Nano. (2019) 13:3555–72. 10.1021/acsnano.9b00033 30860808 PMC7899146

[50] Pala R Mohieldin AM Shamloo K Sherpa RT Kathem SH Zhou J. Personalized nanotherapy by specifically targeting cell organelles to improve vascular hypertension. Nano Lett. (2019) 19:904–14. 10.1021/acs.nanolett.8b04138 30582331 PMC7899193

