# Supplementary material for: Arrhythmogenic Hearts in PKD2 Mutant Mice Are Characterized by Cardiac Fibrosis, Systolic, and Diastolic Dysfunctions
Source: Front Cardiovasc Med. 2021 Nov 26;8:772961. doi: 10.3389/fcvm.2021.772961 (PMC8661014; doi:10.3389/fcvm.2021.772961)
Supplement: Supplementary file 1 [file Table_1.pdf]

SUPPLEMENT TABLES

Arrhythmogenic hearts in PKD2 mutant mice are characterized by cardiac fibrosis, systolic and diastolic dysfunctions.

Farideh Amirrad, M.D.[1,2], Rajasekharreddy Pala, Ph.D.[1], Kiumars Shamloo, M.D., Ph.D.[1],
Brian S. Muntean, Ph.D.[3], Surya M. Nauli, Ph.D.[1,2]

[1]Department of Biomedical and Pharmaceutical Sciences, Chapman University, Irvine, CA 92618
[2]Department of Medicine, University of California Irvine, Orange, CA 92868
[3]Department of Pharmacology and Toxicology, Medical College of Georgia, Augusta, Augusta University, GA 30912

Corresponding author:
Surya M. Nauli
Chapman University
University of California Irvine
9401 Jeronimo Road.
Irvine, CA 92618-1908
Tel: 714-516-5480
Fax: 714-516-5481
Email: nauli@chapman.edu; snauli@uci.edu

Supplement Table 1. Effects of (+) and (-) inotropic on heart functions

	MyH6•Pkd2$^{WT/WT}$			*MyH6•Pkd2$^{flox/flox}$*		
	**Control**	**Adrenalin**	**Diltiazem**	**Control**	**Adrenalin**	**Diltiazem**
**HR (beat/min)**	131±34	143±31	97±28	127±22	156±16	94±9
**ESPVR (mmHg/µL)**	4.8±0.3	14.6±1.4	1.8±0.1	6.1±0.5	7.5±0.5	5.5±0.4
**EDPVR (mmHg/µL)**	0.14±0.01	0.14±0.01	0.14±0.01	0.14±0.01	0.15±0.03	0.14±0.01
**dP/dtmax (mmHg/s)**	5677±271	14538±1504	2945±1616	7517±211	15586±997	3870±1294
**dP/dtmin (mmHg/s)**	-1813±89	-3299±1768	-1472±47	-1608±64	-3111±557	-1000±686
**LV Pmax (mmHg)**	52.6±2.5	72.7±7.5	32.7±1.8	69.6±2.0	77.9±5.0	43.0±14.4
**LV ESP (mmHg)**	39.4±1.9	54.5±5.6	24.6±1.4	52.2±1.5	58.5±3.7	32.0±10.8
**LV EDP (mmHg)**	5.6±0.3	5.5±0.3	5.5±0.1	5.0±0.2	5.2±0.9	3.7±1.3
**LV ESV (µL)**	9.8±0.3	6.4±1.4	18.9±3.15	11.6±0.8	10.4±0.2	10.6±0.8
**LV EDV (µL)**	40.3±0.1	40.1±0.2	40.1±0.04	35.5±0.1	35.4±0.2	35.4±0.2
**SV (µL)**	31.1±0.2	34.7±0.3	23.7±1.2	23.9±0.8	25.0±0.1	24.8±0.9
**SW (mmHg•µL)**	1434±32	2262±364	578±240	1542±53	1816±28	975±57
**EF (%)**	76.7±0.5	86.3±0.9	58.8±3.0	67.2±2.2	70.6±0.4	70.1±2.3
**CO (µL/min)**	4039±87	5269±50	2800±142	3032±105	3896±18	2230±85

HR, heart rate; ESPVR and EDPVR, end-systolic and end-diastolic pressure volume relation, respectively; dP/dtmax and dP/dtmin, maximum rate of left ventricle (LV) pressure rise and fall, respectively; Pmax, systolic pressure; ESP, end-systolic pressure; EDP, end-diastolic pressure; ESV, end-systolic volume; EDV, end-diastolic volume; SV, stroke volume; SW, stroke work; EF, ejection fraction; CO, cardiac output.

Supplement Table 2. Effects of different preloads on heart functions

	MyH6•Pkd2$^{WT/WT}$			*MyH6•Pkd2$^{flox/flox}$*		
	**5 mmHg**	**10 mmHg**	**15 mmHg**	**5 mmHg**	**10 mmHg**	**15 mmHg**
**HR (beat/min)**	125±20	131±26	120±22	127±22	135±19	131±21
**ESPVR (mmHg/µL)**	4.9±0.08	3.6±0.04	3.0±0.04	6.1±0.5	5.7±0.2	6.0±0.2
**EDPVR (mmHg/µL)**	0.13±0.01	0.15±0.03	0.13±0.02	0.14±0.01	0.12±0.01	0.12±0.01
**dP/dtmax (mmHg/s)**	5452±16	6069±125	6502±69	7517±211	7548±224	7541±188
**dP/dtmin (mmHg/s)**	-1656±103	-2205±146	-2050±118	-1608±64	-1307±101	-1325±111
**LV Pmax (mmHg)**	50.5±0.2	56.2±1.2	60.2±0.6	69.6±2.0	69.9±2.1	69.8±1.7
**LV ESP (mmHg)**	37.9±0.1	42.1±0.9	45.2±0.5	52.2±1.5	52.4±1.6	52.4±1.3
**LV EDP (mmHg)**	5.1±0.1	6.8±1.3	6.3±0.8	5.0±0.2	4.0±0.3	4.1±0.3
**LV ESV (µL)**	10.3±0.1	15.2±0.1	20.1±0.1	11.6±0.8	12.2±0.6	11.8±0.4
**LV EDV (µL)**	40.6±0.3	45.3±0.1	50.3±0.1	35.5±0.1	35.0±0.1	35.2±0.1
**SV (µL)**	30.2±2.7	30.1±1.3	30.1±0.1	23.9±0.8	23.0±0.6	23.5±0.3
**SW (mmHg•µL)**	1372±26	1485±26	1623±39	1542±53	1505±41	1545±21
**EF (%)**	74.6±0.2	66.4±0.3	59.9±0.1	67.2±2.2	65.0±1.7	66.7±1.0
**CO (µL/min)**	3799±42	3933±22	3626±8	3032±105	3089±77	3069±44

HR, heart rate; ESPVR and EDPVR, end-systolic and end-diastolic pressure volume relation, respectively; dP/dtmax and dP/dtmin, maximum rate of left ventricle (LV) pressure rise and fall, respectively; Pmax, systolic pressure; ESP, end-systolic pressure; EDP, end-diastolic pressure; ESV, end-systolic volume; EDV, end-diastolic volume; SV, stroke volume; SW, stroke work; EF, ejection fraction; CO, cardiac output.
