# Supplementary material for: Arrhythmogenic Hearts in PKD2 Mutant Mice Are Characterized by Cardiac Fibrosis, Systolic, and Diastolic Dysfunctions
Source: Front Cardiovasc Med. 2021 Nov 26;8:772961. doi: 10.3389/fcvm.2021.772961 (PMC8661014; doi:10.3389/fcvm.2021.772961)
Supplement: Supplementary file 2 [file Data_Sheet_1.pdf]

SUPPLEMENT MATERIALS

Arrhythmogenic hearts in PKD2 mutant mice are characterized by cardiac fibrosis, systolic and diastolic dysfunctions.

Farideh Amirrad, M.D.[1,2], Rajasekharreddy Pala, Ph.D.[1], Kiumars Shamloo, M.D., Ph.D.[1], Brian S. Muntean, Ph.D.[3], Surya M. Nauli, Ph.D.[1,2]

[1]Department of Biomedical and Pharmaceutical Sciences, Chapman University, Irvine, CA 92618
[2]Department of Medicine, University of California Irvine, Orange, CA 92868
[3]Department of Pharmacology and Toxicology, Medical College of Georgia, Augusta, Augusta University, GA 30912

Corresponding author:
Surya M. Nauli
Chapman University
University of California Irvine
9401 Jeronimo Road.
Irvine, CA 92618-1908
Tel: 714-516-5480
Fax: 714-516-5481
Email: nauli@chapman.edu; snauli@uci.edu

Supplement Figure Legends

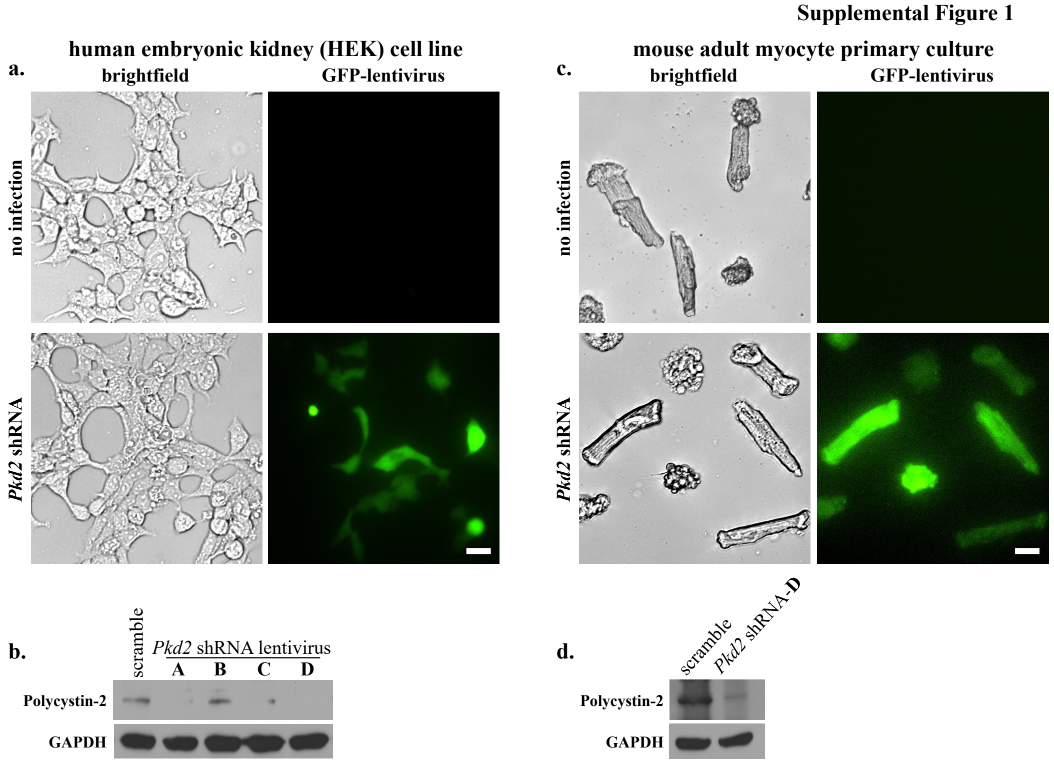

Supplement Figure 1. Confirmation of *Pkd2* knockdown.
Kidney cells were used to test infection efficiency of the lentivirus carrying *shRNA-GFP* (**a**), and lysates from cells infected with scramble (control) or various *Pkd2* shRNAs (A, B, C, and D) were analyzed for *Pkd2* knockdown efficiency (**b**). The *Pkd2-shRNA-D* was used to analyze infection (**c**) and knockdown (**d**) efficiencies in primary myocytes. Bar=10 μm.

Supplement Figure 2

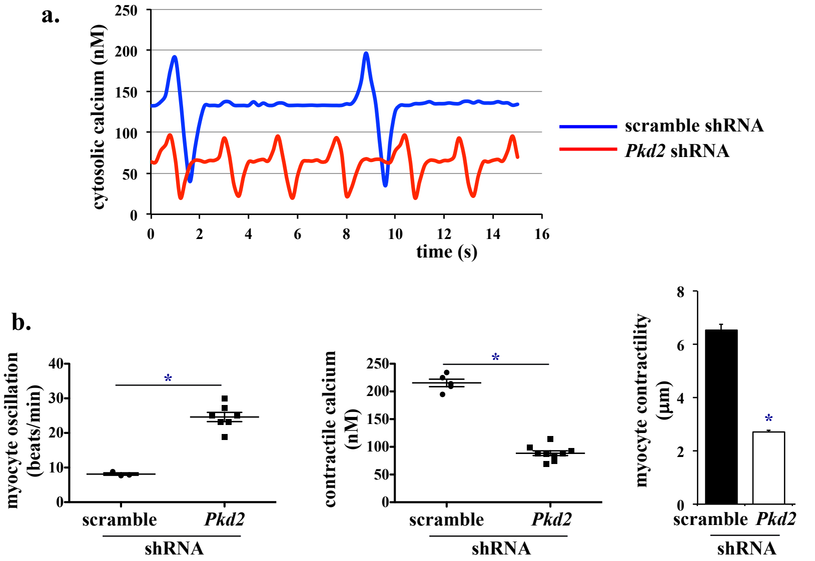

<u>**Supplement Figure 2.** Knockdown *Pkd2* mouse myocytes.</u>
The *Pkd2* knockdown in myocytes are characterized by low contractile calcium, high calcium oscillation frequency and decreased contractile function. Peak-to-peak intervals for cytosolic calcium and heart contraction were measured to determine heart oscillation frequency. Analyzing peak-to-valley amplitude of the oscillations determined heart oscillation magnitude. Representatives of contractile calcium oscillation were taken from a single myocyte (**a**). Contractile frequency and calcium were then averaged. Contractile function was measured by the difference in myocyte length during contraction and relaxation (**b**). Contractility of *Pkd2* knockout in *Pkd2* knockdown in myocytes was significantly decreased compared to the corresponding control groups. Asterisks indicate significant difference from the control group ($p<0.05$). N=5-7 for each group.

Supplement Figure 3

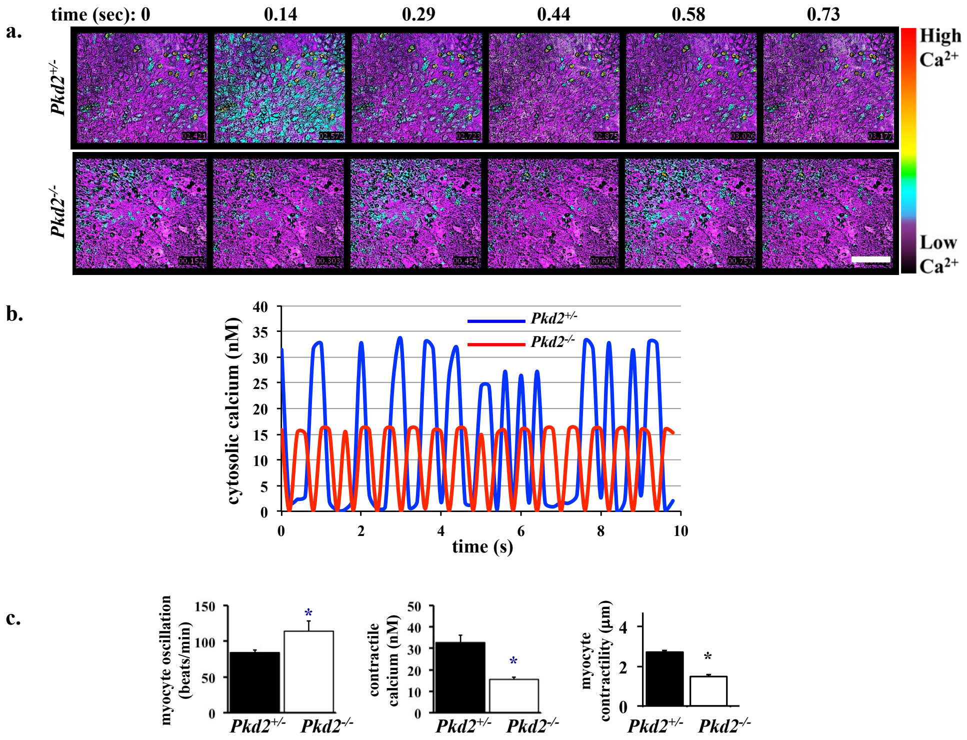

Supplement Figure 3. Knockout *Pkd2* mouse myocytes.

The *Pkd2* knockout in myocytes are characterized by low contractile calcium, high calcium oscillation frequency and decreased contractile function. Peak-to-peak intervals for cytosolic calcium and heart contraction were measured to determine heart oscillation frequency. Analyzing peak-to-valley amplitude of the oscillations determined heart oscillation magnitude. Intracellular myocyte calcium of myocytes was studied and pseudocolored (**a**). Color bar indicates myocyte calcium level, where black-purple and yellow-red colors represent low and high calcium levels, respectively. Representatives of contractile calcium oscillation were taken from a single myocyte (**b**). Contractile frequency and calcium were averaged (**c**). N=4 for each group. Bar=100 mm

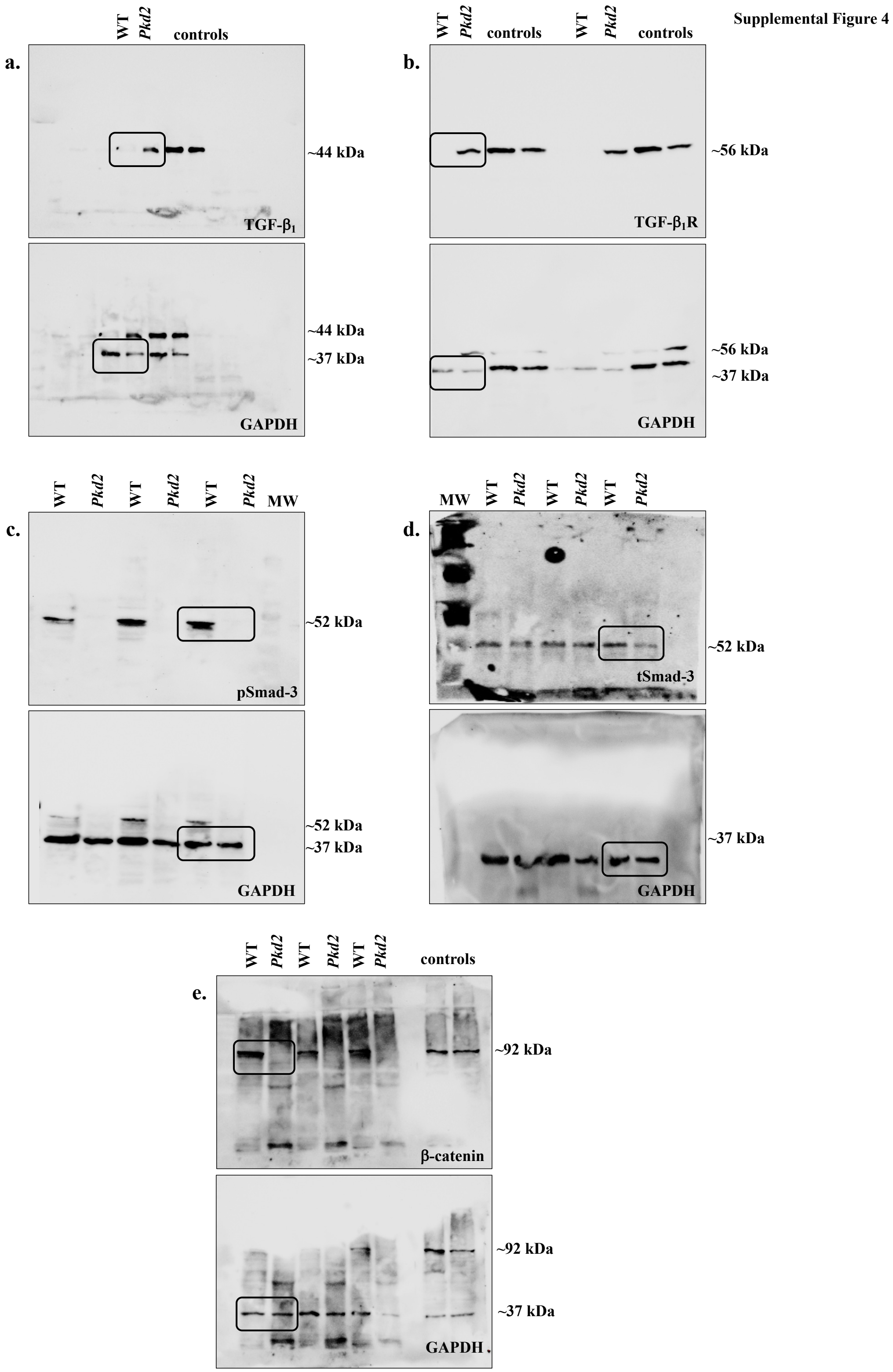

Supplement Figure 4. Evaluation of different mediators.
Expressions of TGF-β_1, TGF-β_1 receptor, pSMAD3, tSMAD3, and β-catenin were analyzed in hearts of wild-type (WT) and *Pkd2*-KO (*Pkd2*) mice. The original uncropped blots are shown. Boxes are cropped and presented in Figure 7.

Supplemental Figure 5

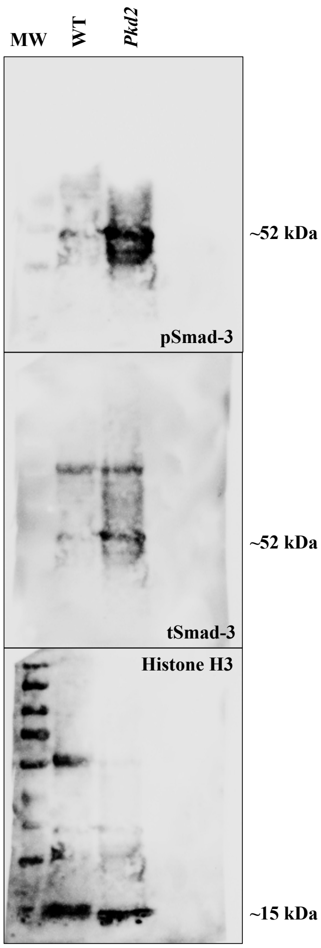

<u>**Supplement Figure 5.** Evaluation of SMAD3 in the nucleus.</u>

Heart tissue lysates were used to evaluate pSMAD3 and tSMAD3 in the wild-type (WT) and *Pkd2*-KO (*Pkd2*) nucleus. Results indicated a higher localization of pSMAD3 in the *Pkd2*-KO nucleus compared to WT.

Supplement Video Legends

Video 1. Intracellular calcium oscillation in scrambled shRNA myocyte.
Primary mouse myocytes were subjected to a non-specific scrambled shRNA lentivirus for 48 hours in standard culture conditions. Intracellular calcium was then studied with Fura-2AM. Pseudocoloring indicates calcium range from low (purple), intermediate (blue-green), and high (yellow-red). Time is indicated in seconds:milliseconds.

Video 2. Intracellular calcium oscillation in *Pkd2* shRNA myocyte.
Primary mouse myocytes were subjected to a *Pkd2* specific shRNA lentivirus for 48 hours in standard culture conditions. Intracellular calcium was then studied with Fura-2AM. Pseudocoloring indicates calcium range from low (purple), intermediate (blue-green), and high (yellow-red). Time is indicated in seconds:milliseconds.

Video 3. Intracellular calcium oscillation in $Pkd2^{+/-}$ cardioacmyocytes
Cardiomyocytes were cultured from the dissected hearts. Frequency of spontaneous myocyte contractions was observed under phase microscopy. Intracellular calcium was studied utilizing Fura-2AM. Pseudocoloring indicates calcium range from low (purple), intermediate (blue-green), and high (yellow-red). Time is indicated in seconds:milliseconds.

Video 4. Intracellular calcium oscillation in $Pkd2^{-/-}$ cardioacmyocytes
Cardiomyocytes were cultured from the dissected hearts. Frequency of spontaneous myocyte contractions was observed under phase microscopy. Intracellular calcium was studied utilizing Fura-2AM. Pseudocoloring indicates calcium range from low (purple), intermediate (blue-green), and high (yellow-red). Time is indicated in seconds:milliseconds.

Video 5. The isolated working heart system shown for a mouse heart
Video was originally taken with iPhone11 to help envision the experimental setting. Parts of the working heart system are labelled accordingly.
